# Characterization of proteins present in the biofilm matrix and outer membrane vesicles of *Histophilus somni* during iron-sufficient and iron-restricted growth: identification of potential protective antigens through *in silico* analyses

**DOI:** 10.1128/mbio.00644-25

**Published:** 2025-04-17

**Authors:** Yue-Jia Lee, Mohd Abdullah, Yung-Fu Chang, Habeeb Al Sudani, Thomas J. Inzana

**Affiliations:** 1 Department of Veterinary Biomedical Sciences, College of Veterinary Medicine, Long Island University 42805 , Brookville, New York, USA; 2 Institute of Food Science and Technology, National Taiwan University, Taipei, Taiwan; 3 Department of Population Medicine and Diagnostic Sciences, College of Veterinary Medicine, Cornell University, Ithaca, New York, USA; 4 Cancer Center, Cold Spring Harbor Laboratory 2595, Cold Spring Harbor, New York, USA; Fondazione Biotecnopolo di Siena, Siena, Italy

**Keywords:** *Histophilus somni*, outer membrane vesicles, biofilm matrix, iron acquisition, reverse vaccinology, protective antigen

## Abstract

There is limited efficacy in vaccines currently available to prevent some animal diseases of bacterial origin, such as bovine respiratory disease caused by *Histophilus somni*. Protective efficacy can potentially be improved if bacterial antigens that are expressed in the host are included in vaccines. During *H. somni* infection in the bovine host, biofilms become established, and the availability of essential iron is restricted. To investigate further, the protein composition of spontaneously released outer membrane vesicles (OMVs) during iron-sufficient and iron-restricted growth and the proteins expressed in the biofilm matrix were analyzed and compared. Proteomic analysis revealed a dramatic physiological change in *H. somni* as it transitioned from the planktonic form to the biofilm mode of growth. All transferrin-binding proteins (Tbps) previously identified in *H. somni* were detected in the OMVs, suggesting that OMVs could induce antibodies to these proteins. Two TbpA-like proteins and seven total proteins were present in the OMVs only when iron was restricted, indicating the expression of these Tbps was differentially regulated. More proteins associated with quorum-sensing (QS) signaling were detected in the biofilm matrix compared with proteins in the OMVs, supporting a link between QS and biofilm formation. Proteins ACA31267.1 (OmpA) and ACA32419.1 (TonB-dependent receptor) were present in the OMV and biofilm matrix and predicted to be potential protective antigens using an immuno-bioinformatic approach. Overall, the results support the development of novel vaccines that contain OMVs obtained from bacteria grown to simulate the *in vivo* environment, and possibly biofilm matrix, to prevent diseases caused by bacterial pathogens.

**IMPORTANCE:**

Bovine respiratory disease (BRD) is the most economically important disease affecting the cattle industry. Available BRD vaccines consist of killed bacteria but are not very effective. Poor vaccine efficacy may be because the phenotype of bacteria in the host differs from the phenotype of cultured bacteria. Following host infection, virulent bacteria can express transferrin-binding proteins (Tbps) not expressed in culture medium but are required to sequester iron from host proteins. During chronic infections, such as BRD, bacteria can form a biofilm consisting of novel protein and polysaccharide antigens. The unique proteins expressed on outer membrane vesicles (OMVs) of *Histophilus somni* (a BRD pathogen) in the absence of iron and as a biofilm were identified and characterized. At least two TbpA-like proteins were expressed in OMVs only under iron-limiting conditions. Quorum-sensing-associated proteins were identified in the *H. somni* biofilm matrix. *In silico* analysis identified potential protein targets for vaccine development.

## INTRODUCTION

*Histophilus somni* is one of the predominant bacterial agents responsible for bovine respiratory disease (BRD) but is also responsible for systemic diseases, such as septicemia, myocarditis, thrombotic-meningoencephalitis, arthritis, reproductive failure, and others (1). *H. somni* is an opportunistic pathogen and only resides in the respiratory and genital mucosal membranes of ruminants (2). Following periods of stress, such as prior viral infection, climate extremes, crowding, shipping, etc., the bacteria may evade innate host defenses and are thought to enter the lower respiratory tract and then the bloodstream (3). Virulent strains possess a wide variety of virulence factors that enable them to evade host innate and adaptive defense mechanisms. Such virulence factors include lipooligosaccharide (LOS) (4), novel matrix components responsible for biofilm formation (5), survival inside phagocytic cells (6), and expression of immunoglobulin binding protein (IbP). IbP also contributes to adherence to host sites and contains a cytotoxic Fic motif (7). In addition, the attachment of sialic acid to the LOS and biofilm exopolysaccharide (EPS), antigenic mimicry of the LOS with host antigens, and attachment of phase-variable phosphorylcholine to the LOS further contribute to the evasion of host defenses by *H. somni* (8–12). Furthermore, during infection of the host, *H. somni* must express iron-binding proteins in order to sequester iron from ruminant transferrin. These antigens are not expressed during growth in rich medium, where iron is readily available (13).

The hallmark of *H. somni* diseases is vasculitis and thrombus formation (3). Inflammation and cytotoxicity of the vascular endothelium are proposed to be responsible for bacteremia and dissemination of the bacteria to various tissues (7). The lipid A component of the LOS, possibly peptidoglycan, and cytotoxicity due to Fic all contribute to inducing an inflammatory response that is largely responsible for disease manifestation, including cellular apoptosis and platelet aggregation (3, 14, 15).

The natural state of growth for *H. somni* is a biofilm, which also forms during chronic infection (5), and is an excellent model for biofilm-related infections. The biofilm matrix consists largely of a galacto-mannan EPS, which is not produced during planktonic growth (12). However, commercial vaccines are made from bacteria grown planktonically and lack factors utilized by and expressed on the surface of the bacteria *in vivo*, such as sialic acid (11) and iron-binding proteins (13). Furthermore, half of the bacterial genome is differentially expressed when the bacteria form a biofilm compared to planktonic growth (16). Therefore, the biofilm and surface antigens expressed by opportunistic mucosal pathogens in the host may be very different from those present in vaccines prepared from planktonically grown bacteria. Bacterial cells in commercial vaccines do not express cell surface transferrin binding proteins (Tbps) because these proteins are only induced in the absence of iron, which is readily present in culture medium. In order to grow and survive in the host, bacteria must be able to sequester iron from host sources, such as transferrin and lactoferrin. In addition, the EPS and many biofilm matrix proteins are not present in vaccines prepared from planktonically grown bacteria. Outer membrane vesicles (OMVs) are a rich source of surface antigens expressed by gram-negative bacteria, are relatively simple to prepare, and have been shown to induce protective immunity against bacterial diseases of animals and humans (17, 18). Our aim was to identify novel proteins present in OMV during growth in the absence of iron and in the biofilm matrix, as well as to identify protein(s) that could act as protective antigen(s) against *H. somni*.

## RESULTS

### Novel proteins are present in *H. somni* OMV during planktonic growth in the absence of iron and in the biofilm matrix

**Fig 1:**
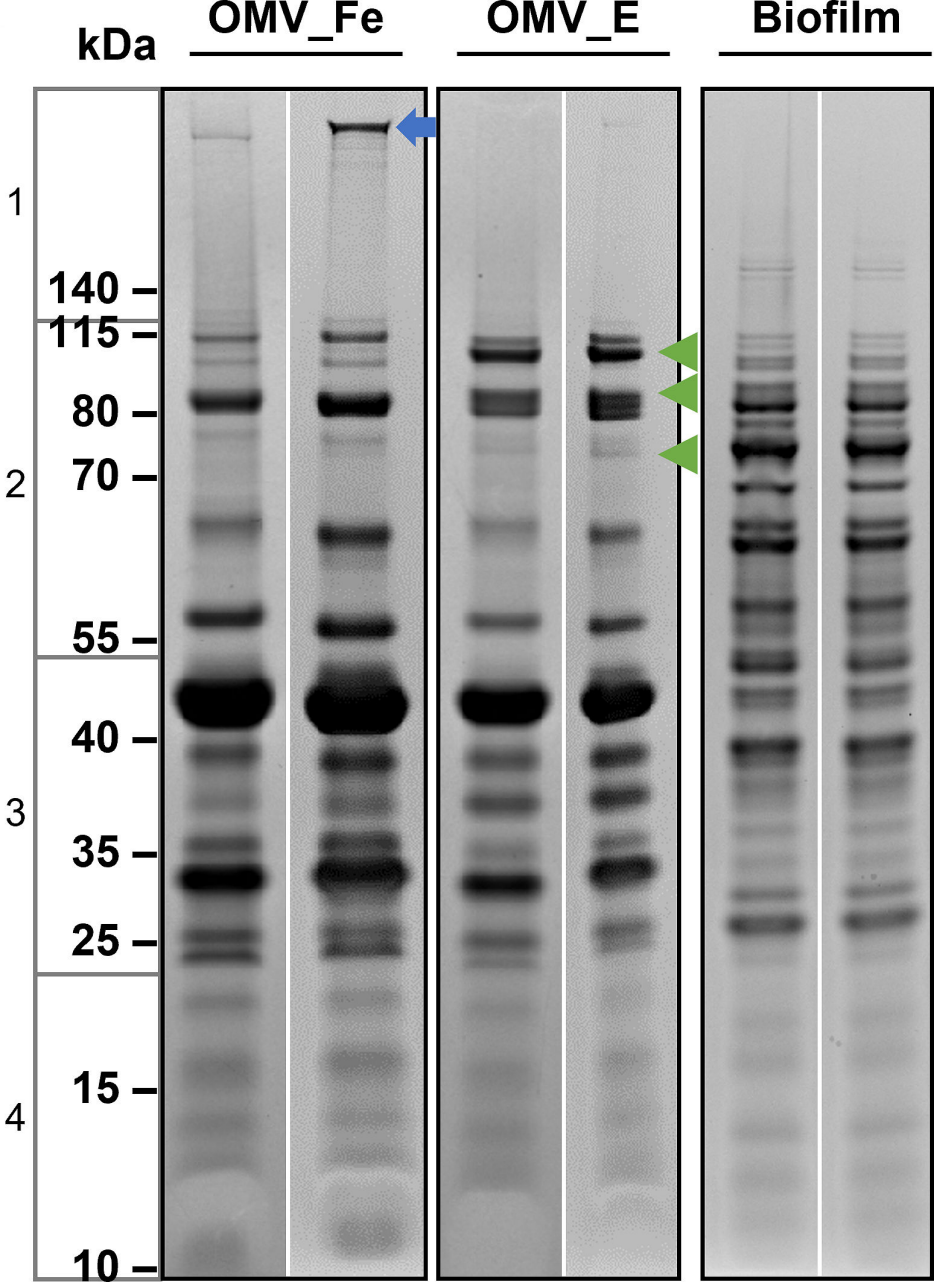
Protein profiles of the OMVs and biofilm matrix in *H. somni* strain 2336. Duplicate protein samples were obtained from the OMVs of *H. somni* grown under Fe(NO_3_)_3_ treatment (OMV_Fe, lanes 1 and 2), EDDHA treatment (OMV_E, lanes 3 and 4), or from the biofilm matrix of *H. somni* grown under static conditions for biofilm formation (Biofilm, lanes 5 and 6). The protein ladder is shown on the left side and is separated by four boxes to indicate the splitting of the gel lane for proteomic analysis. Gel images were segmented for illustration purposes: white space was increased between duplicate protein samples, and different growth conditions were marked with black boxes. The blue arrow points to putative protein IbpA, a documented protective antigen against *H. somni* diseases (this protein is also present in the biofilm but is normally only identified by Western blotting, as previously reported [19]). Green arrows point to Tbps that were induced by EDDHA treatment: TbpA, 109.6 kDa; TbpA2, 84.6 kDa; and TbpB, 71.3 kDa, from top to bottom.

OMVs were released from *H. somni* in large numbers, primarily egg-shaped, and in highly variable sizes that differed as much as 10-fold (Fig. S1). There did not appear to be any differences in the size or shape of OMV from bacteria grown in iron-rich or iron-deprived medium (data not shown). The proteins recovered from (i) OMVs released under Fe (NO_3_)_3_ treatment, (ii) OMVs released during growth in medium containing ethylenediamine-N,N′-bis((2-hydroxyphenyl)acetic acid) (EDDHA) (Santa Cruz Biotechnology, Dallas, TX, USA) treatment, or (iii) total proteins recovered from the biofilm matrix of *H. somni* were examined by sodium dodecyl sulfate-polyacrylamide gel electrophoresis (SDS-PAGE). These OMV proteins are located primarily on the bacterial surface and may therefore interact with host cells and mediators of the immune system. Most of the proteins from all tested groups were distributed predominately in the size range from 25 to 115 kDa (Fig. 1). However, many of the proteins present in the biofilm matrix were very different from the proteins in OMV, regardless of iron availability, particularly the major outer membrane proteins (~45 and 32 kDa). Therefore, it is likely that few OMV proteins were present in the biofilm matrix.

**Fig 2:**
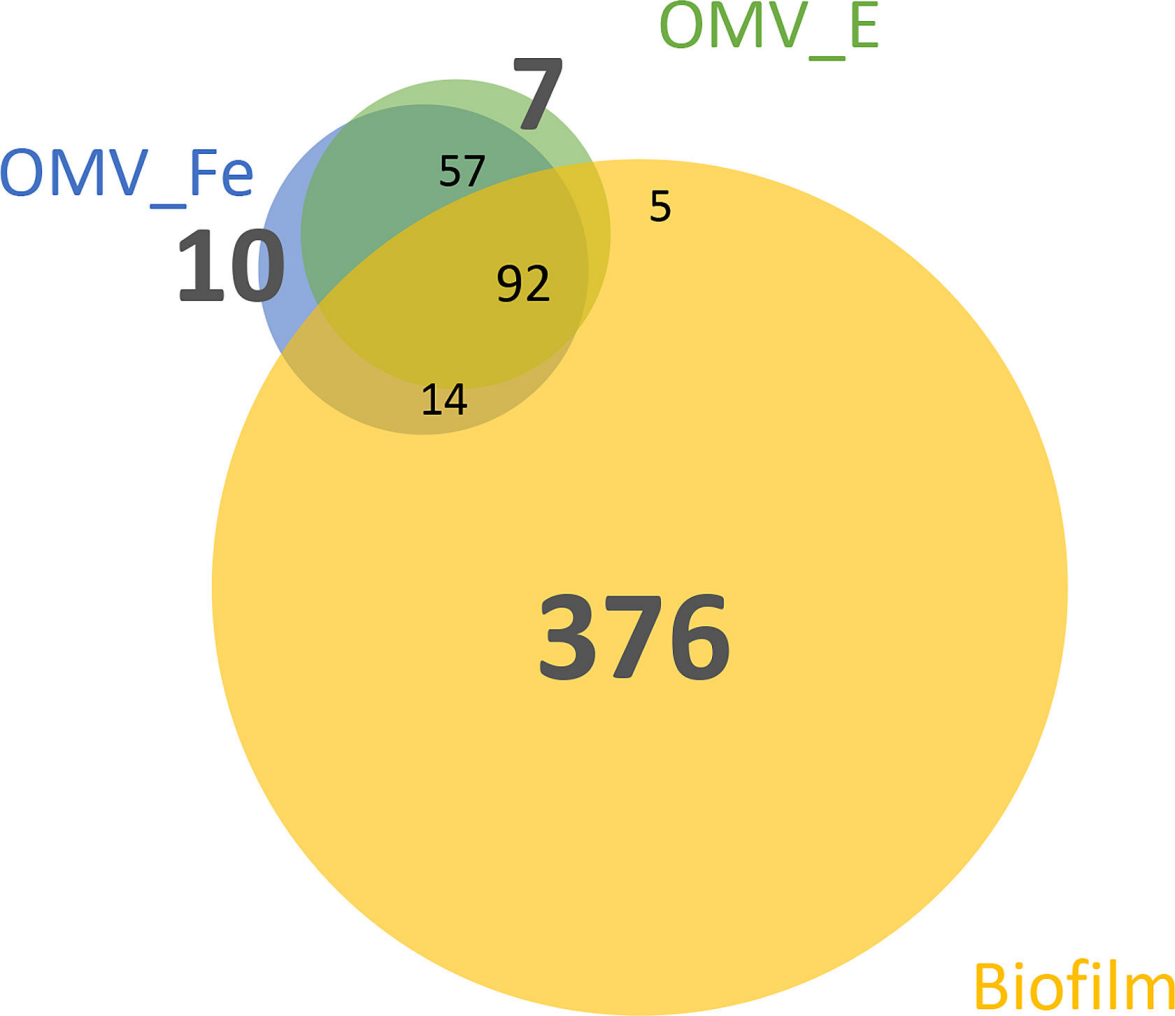
Venn diagram of the presence of proteins overlapping in different biological samples. OMV_Fe: proteins in OMVs collected under Fe(NO_3_)_3_ treatment (an iron-sufficient condition); OMV_EDDHA: proteins in OMVs collected under EDDHA treatment (an iron-deficient condition); Biofilm: proteins in biofilm matrix of *H. somni*. A protein was counted as present when it was found in both biological replicates of one growth condition. The size of each circle is proportional to the protein count.

To identify those proteins that were exclusively present during iron-restricted growth or biofilm formation, LC/MS-MS was performed on the proteins extracted from SDS-PAGE gels (Table S1). The number of proteins (487) detected in the biofilm matrix was much greater than those in the OMV samples (173 and 161 for ferric nitrate-treated and EDDHA-treated OMVs, respectively). Only 10 and 7 proteins were exclusively identified in ferric nitrate-treated and EDDHA-treated OMVs, respectively, and 376 proteins were exclusively present in biofilm matrix samples. Ninety-two common proteins were identified under the three different growth conditions: OMV_Fe, OMV_EDDHA, and Biofilm (Fig. 2). The substantial number of unique proteins in the biofilm matrix demonstrated a dramatic physiological change in *H. somni,* as it switched from the planktonic form to the biofilm mode of growth.

**Fig 3:**
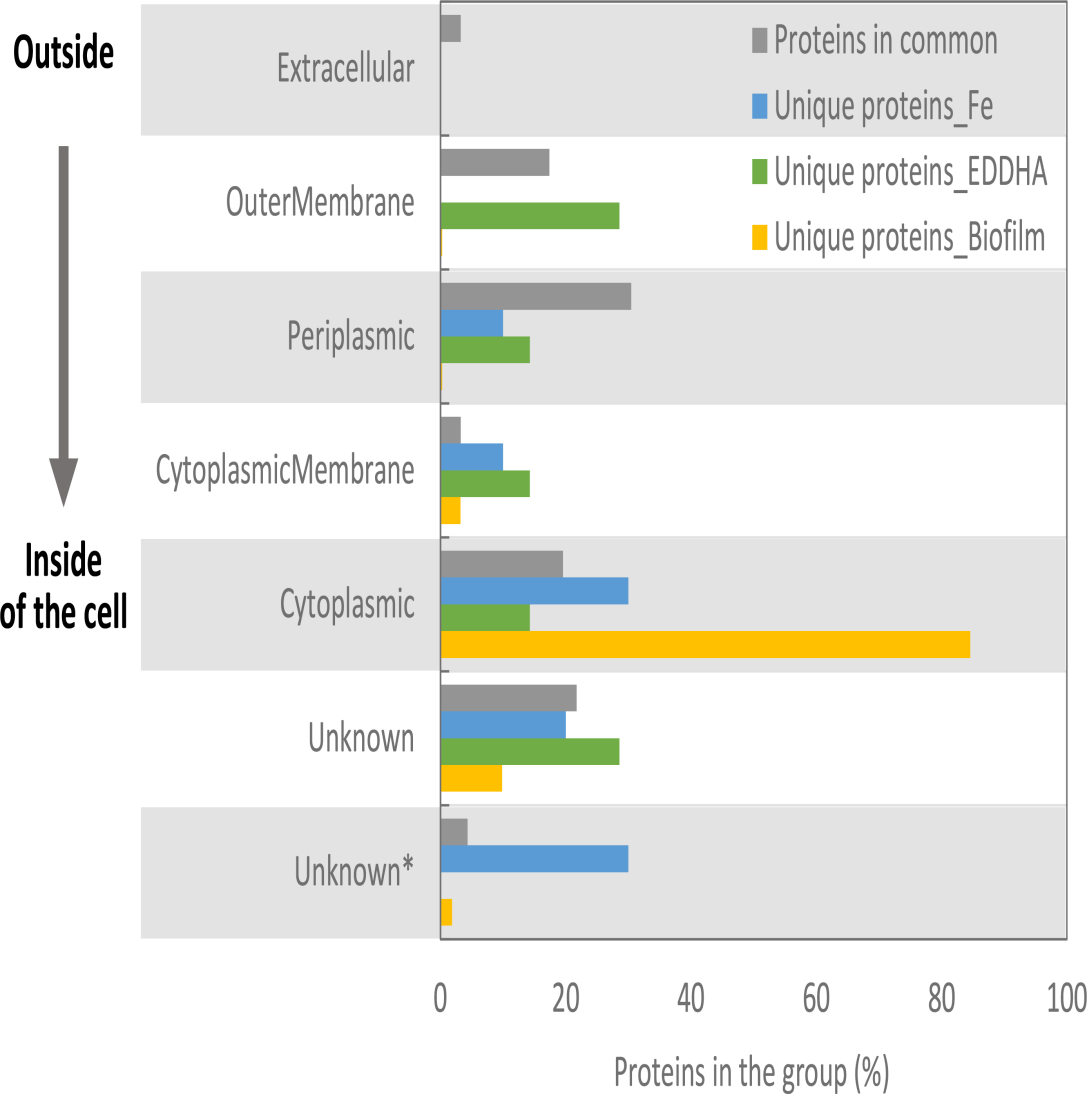
Predicted localization of proteins in three different biological samples. Common and unique proteins identified in OMVs with ferric nitrate treatment (Fe), EDDHA treatment (EDDHA), or in the biofilm matrix (Biofilm) were assigned to seven groups (top to bottom: outside to inside of the cell) of cellular localization based on the scores from PSORTb analysis. Proteins assigned to the Unknown group lack a predicted localization. Proteins assigned to the Unknown group labeled with an asterisk (*) show a distribution of localization scores favoring two sites. The proportion for each localization group was calculated as follows: (the count of proteins assigned to the group for localization)/(the count of total proteins identified in both replicates of the growth condition).

Based on PSORTb analysis, the distribution of distinct proteins identified in OMVs and the biofilm matrix varied throughout different cellular regions (Fig. 3). Among 10 proteins exclusively identified in ferric nitrate-treated OMVs, one protein was predicted to be in the periplasm, one in the cytoplasmic membrane, three proteins in the cytoplasm, and two were assigned to the Unknown group. While no proteins were assigned to the extracellular or outer membrane sites, three proteins assigned to the Unknown* group were YadA domain proteins, and they exhibited a distribution of localization scores that favored extracellular (6.04) and outer membrane sites (3.6). Among seven proteins exclusively identified in EDDHA-treated OMVs, two proteins were predicted to be in the outer membrane, one was in the periplasm, one was in the cytoplasmic membrane, one was in the cytoplasm, and two were assigned to the Unknown group. Of 376 unique proteins identified in the biofilm matrix, the majority (318) were predicted to be in the cytoplasm, 1 was in the outer membrane, 1 was in the periplasm, 12 were in the cytoplasmic membrane, and 37 were assigned to the Unknown group. Out of the seven proteins in the Unknown* group, one protein exhibited localization scores favoring extracellular (6.04) or outer membrane sites (3.6), two proteins were predicted to have a preference for periplasm (4.99) or cytoplasmic membrane (4.9) sites, and the remaining four proteins were predicted to have preference for the periplasm (4.48) or the cytoplasm (5.41).

Of 92 common proteins identified under all three growth conditions (Table S2), 3 proteins were predicted to be extracellular, 16 were predicted to be in the outer membrane, 28 were predicted to be periplasmic, 3 were in the cytoplasmic membrane, 18 were predicted to occur in the cytoplasm, and the localization of 20 proteins was unknown. For the remaining four proteins in the Unknown* group, two proteins exhibited localization scores favoring periplasm (5.81) or outer membrane (3.9) locations (ACA32109.1 and ACA32359.1), one protein in the periplasm (4.99) or cytoplasmic membrane (4.9) (CAY37763.1), and one protein in the periplasm (4.48) or cytoplasm (5.41) (ACA31503.1). In summary, a higher proportion of proteins identified in the OMV samples were predicted to be located in extracellular or outer membrane sites, compared to the proteins unique to the biofilm matrix.

### Functional annotation of unique proteins in planktonic OMVs during iron-restricted growth and in the biofilm matrix

**Fig 4:**
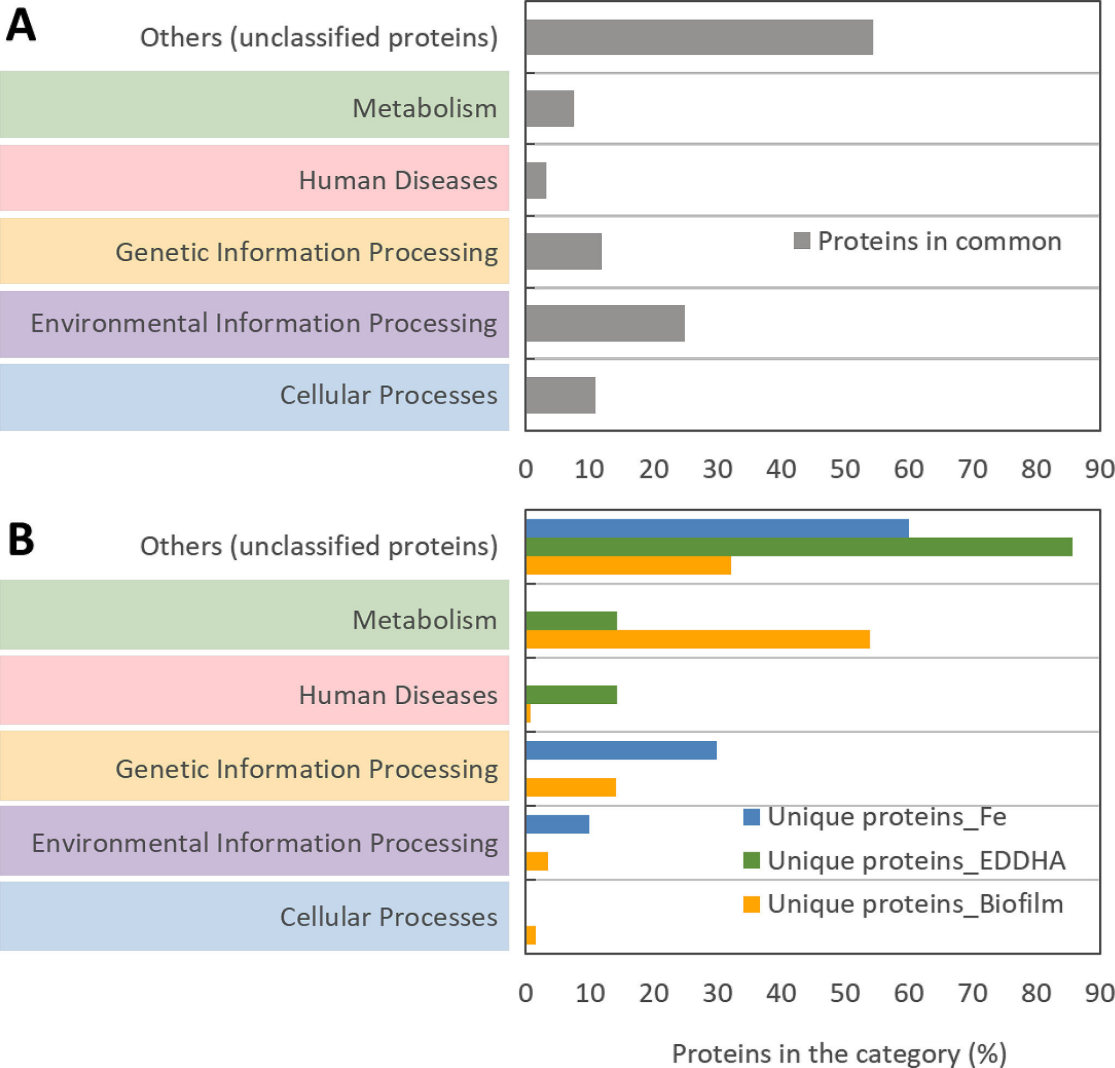
KEGG pathway analysis for the identification of functional categories of proteins expressed under different growth and purification conditions. Common (panel A) and unique (panel B) proteins identified in OMVs with ferric nitrate treatment (Fe), EDDHA treatment (EDDHA), or in the biofilm matrix (Biofilm) were grouped into five main functional categories of biomolecular pathways (shown in the vertical axis). The percentage of proteins in each category was calculated as follows: (the count of proteins assigned to the pathway category)/(the count of total proteins identified in both replicates of the growth condition). The description of seven pathway map categories in the KEGG PATHWAY (https://www.genome.jp/kegg/disease/) database can be found under “Bioinformatic analysis” in “Materials and Methods.” The Human Diseases category of the KEGG PATHWAY database is a collection of disease pathway maps. It contains multifactorial diseases such as cancers, immune system diseases, neurodegenerative diseases, cardiovascular diseases, and metabolic diseases, where known disease genes are marked in red. It also contains infectious diseases, where interacting molecular networks of both pathogens and humans are depicted.

To better decipher the 92 common and distinct proteins identified during the three different conditions, the KEGG pathway mapping server (https://www.genome.jp/kegg/mapper/search.html) was used to classify the proteins into five categories of biomolecular pathways (Fig. 4, panels A and B, respectively; Table S3). These 92 proteins included well-known virulence factors and iron acquisition proteins, such as IbpA (HSM_1489) and TbpA (HSM_0750) (Table S1). The functional distribution of unique proteins made during the three growth conditions was quite different (Fig. 4, panel B). Of seven unique proteins detected in OMVs produced under EDDHA treatment, four could be categorized by the KEGG server (Fig. 4, panel B) and GenBank. Proteins ACA32614.1 and ACA32651.1 (HSM_0931 and HSM_0932, respectively) were both identified as transferrin-binding TonB-dependent receptor proteins (Fig. 1 and 4), indicating that EDDHA successfully restricted iron acquisition in *H. somni* and that these proteins were expressed in OMVs. Other proteins induced by EDDHA treatment included protein ACA30786.1 (HSM_1071), which was identified as penicillin-binding protein 1A (data not shown). Another iron-restricted induced protein was ACA31186.1, which was identified as a nuclease (SNase domain protein). Three other proteins (ACA31510.1, ACA32586.1, and ACA32592.1) were also induced by EDDHA but were assigned as proteins of unknown function.

Of the 376 unique proteins detected in the biofilm matrix, 255 proteins could be classified into KEGG pathway categories (Fig. 4, panel B; Table S3). Five of six proteins likely to be involved in cellular processes were associated with QS, which is a regulatory system controlling multiple biological processes, including biofilm formation (Table S3). These QS-associated proteins included protein-export protein SecB (HSM_0022) (20), Sec translocon accessory complex subunit YajC (HSM_0303) (21), RNA-binding protein Hfq (HSM_1074) (22), phospho-2-dehydro-3-deoxyheptonate aldolase (HSM_1416), and deoxyribose-phosphate aldolase/phospho-2-dehydro-3-deoxyheptonate aldolase (HSM_1948). Common proteins (panel A) identified under all three growth conditions also included four other QS-associated proteins (HSM_0696, HSM_0697, HSM_0788, and HSM_1947). Interestingly, while HSM_1947 and HSM_1948 are in the same operon in the *H. somni* genome, the protein encoded by HSM_1948 was identified only in the biofilm matrix.

### Identification of putative protective antigens through an *in silico* approach

Among the 92 common proteins observed across all three growth conditions, 19 extracellular/outer membrane proteins were chosen for scrutiny regarding their potential to contribute to virulence and their potential as vaccine candidates. Two distinct proteins, ACA31267.1, an OmpA domain protein, and ACA32419.1, a TonB-dependent lactoferrin and transferrin receptor (TpbA), stood out with a percent identity threshold of ≥50 and a percent query coverage of ≥70 when compared against the extensive Virulence Factor Database (VFDB) protein data set. The remaining 17 proteins were considered to have low potential in terms of virulence and were excluded from subsequent analysis. Furthermore, proteins ACA31267.1 and ACA32419.1 have signal peptides with antigenicity scores of 0.72 and 0.63, respectively, indicating their potential to elicit an immune response (Table 1).

**TABLE 1:** *H. somni* outer membrane proteins analyzed using the Virulence Factor Database and characterized for potential immunogenicity

Gene ID	Protein name	Sequence similarity with VFDB^*a*^	Antigenicity (≥0.4)^*b*^	Transmembrane helices (0 or 1)^*b*^	Signal peptide^*b*^
ACA31267.1	OmpA domain protein	Outer membrane protein PomA	0.7237	0	Yes
ACA32419.1	TonB-dependent lactoferrin and transferrin receptor	(tbpA) Transferrin-binding protein 1 precursor	0.6341	0	Yes
ACA31051.1	SmpA/OmlA domain protein	Non-homologous^*c*^	0.6508	0	Yes
ACA31096.1	Peptidoglycan-associated lipoprotein	Non-homologous	0.5490	0	Yes
ACA31171.1	17 kDa surface antigen	Non-homologous	0.8383	0	Yes
ACA31193.1	Porin gram-negative type	Non-homologous	0.7751	0	Yes
ACA31204.1	Outer membrane chaperone Skp (OmpH)	Non-homologous	0.4929	0	Yes
ACA31205.1	Surface antigen (D15)	Non-homologous	0.5844	0	Yes
ACA31604.1	VacJ family lipoprotein	Non-homologous	0.3073	0	Yes
ACA31830.1	Conserved hypothetical protein	Non-homologous	0.5005	0	No
ACA32113.1	Rare lipoprotein B	Non-homologous	0.2663	1	Yes
ACA32145.1	Peptidase M23B	Non-homologous	0.5148	0	Yes
ACA32292.1	Tetratricopeptide TPR_2 repeat protein	Non-homologous	0.3081	0	Yes
ACA32396.1	Organic solvent tolerance protein	Non-homologous	0.6655	0	Yes
CBN71009.1	Unnamed protein product	Non-homologous	0.6327	1	Yes
CBN71110.1	Unnamed protein product	Non-homologous	0.6838	0	No
ACA31239.1	Cysteine protease domain, YopT-type	Non-homologous	0.6752	0	No
ACA31627.1	Sel1 domain protein repeat-containing protein	Non-homologous	0.5733	0	Yes
DAA01283.1	TPA_exp: putative glycerophosphoryl diester phosphodiesterase	Non-homologous	0.5323	0	Yes

^*a*^ Identified as having the most potential for contributing to *H. somni* virulence.

^*b*^ Antigenicity is a quantitative parameter based on amino acid properties. The threshold for antigenicity is ≥0.4. Transmembrane helices refer to the number of helical turns a protein makes in the outer membrane. For transmembrane helices, 0 or 1 refers to the number of turns the protein has across the membrane.

^*c*^ Non-homologous refers to proteins that have very low or no sequence similarity to proteins in the VFDB. Non-homologous does not imply any specific relationship with antigenicity, adhesion, or transmembrane helices.

### Analysis of molecular docking and structural dynamics simulation of select proteins

**Fig 5:**
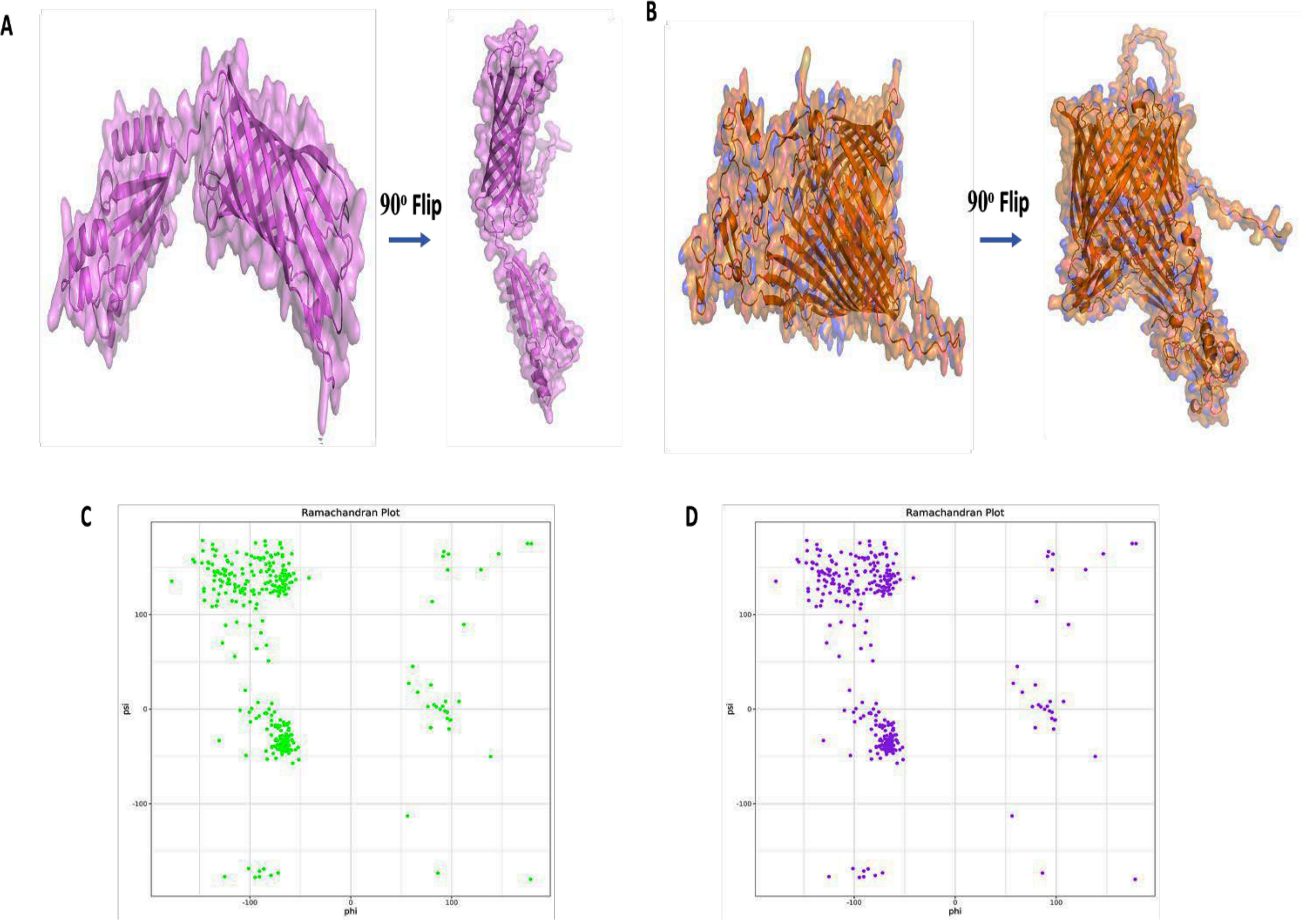
The tertiary structures of two *Histophilus somni* proteins. AlphaFold 3 was used to predict the structures of proteins ACA31267.1 (**A**) and ACA32419.1 (B). The structures demonstrate the spatial arrangement of the protein domains, including helices and beta-sheets, providing insights into their potential functional conformations. A Ramachandran plot shows that 97.7% of amino acid residues of protein ACA31267.1 (**C**) resided within the allowed region, whereas 95% of amino acid residues fell within the permissible region for protein ACA32419.1 (**D**).

The tertiary structures of both proteins were predicted using AlphaFold3 (Fig. 5A and B). The quality of the resulting models was assessed using the Ramachandran plot. In ACA31267.1, approximately 97.7% of the residues fell within the allowed region, while none were in the disallowed region (Fig. 5C). For protein ACA32419.1, about 95% of the residues were in the allowed region, and 2.4% fell into the disallowed region (Fig. 5D).

**Fig 6:**
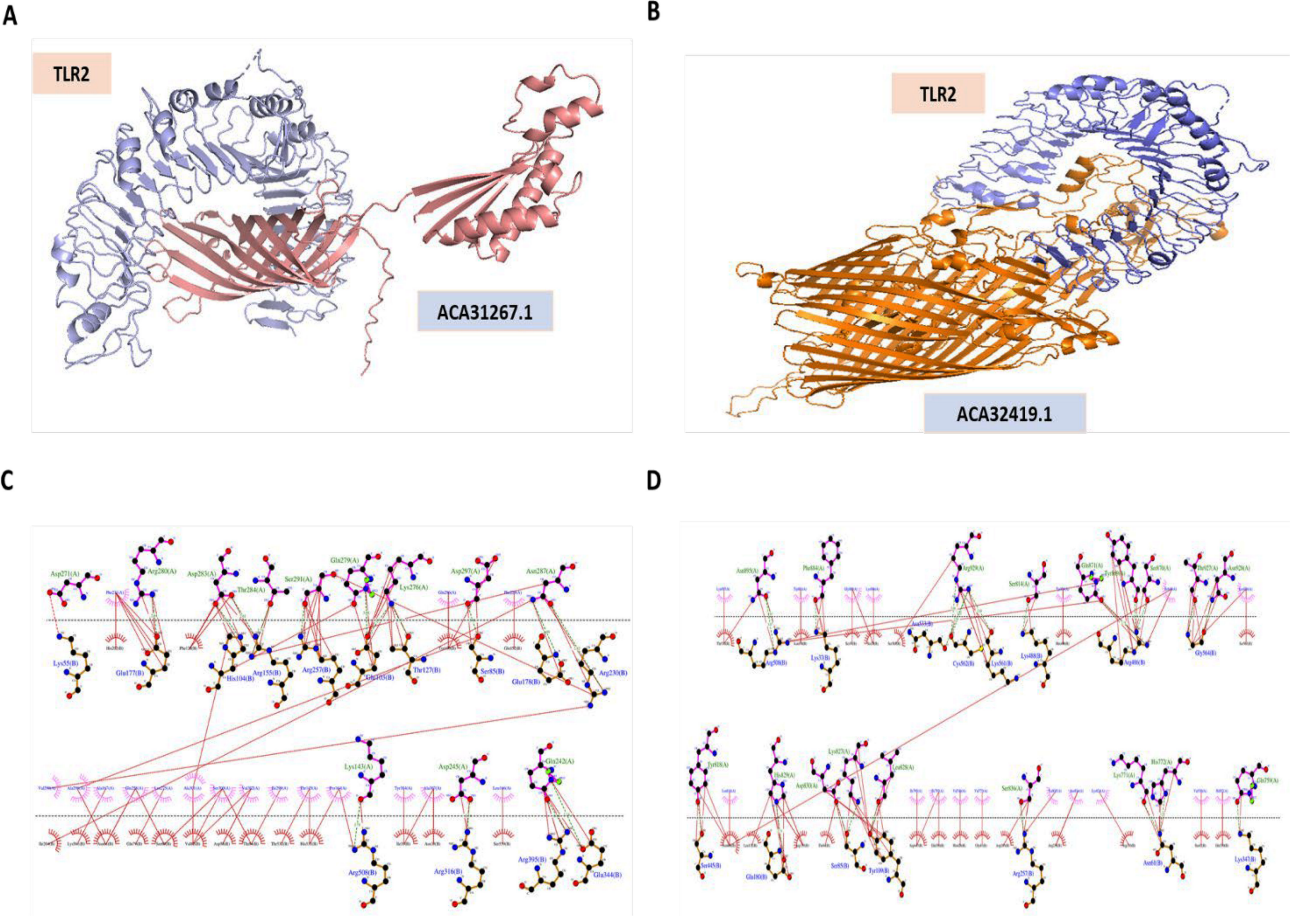
Predicted docking of proteins ACA31267.1 and ACA32419.1 with bovine TLR2. Intramolecular interactions of the *H. somni* proteins ACA31267.1 and ACA32419.1 with bovine TLR2 and the specific amino acid residues that participate in binding between the ligand and TLR2 receptor. (**A**) Docking of ACA31267.1; (**B**) docking of ACA32419.1. (**C**) Intramolecular interaction of ACA31267.1 with TLR2, and (**D**) intramolecular interaction of ACA32419.1 with TLR2.

The interaction between ligands of ACA31267.1 and ACA32419.1 and the bovine TLR2 receptor was investigated using molecular docking (Fig. 6A and B). The resulting complexes were analyzed using LigPlot+ to visualize their intermolecular interactions (Fig. 6C [ACA31267.1] and D [ACA32419.1]). The binding energy between ACA32167.1 and bovine TLR2 was calculated to be −18.4 kcal/mol, indicating a good binding affinity. Similarly, the binding energy of ACA32419.1 was −14.4 kcal/mol, suggesting a favorable interaction (Table 2).

**TABLE 2:** Comprehensive evaluation of the complex formed between ligand and receptor

Tools	Parameter	ACA32419.1	ACA31267.1
HDOCK	Docking score	−491.09	−468.44
HADDOCK	HADDOCK score	−158.4 ± 5.8	−202.8 ± 6.0
	Van der waals energy	−93.5 ± 5.6	−126.8 ± 8.7
	Electrostatic energy	−453.6 ± 37.9	−297.5 ± 20.1
PRODGIGY	Binding energy (Δ*G*) (kcal mol^-1^)	−14.0	−18.4

**Fig 7:**
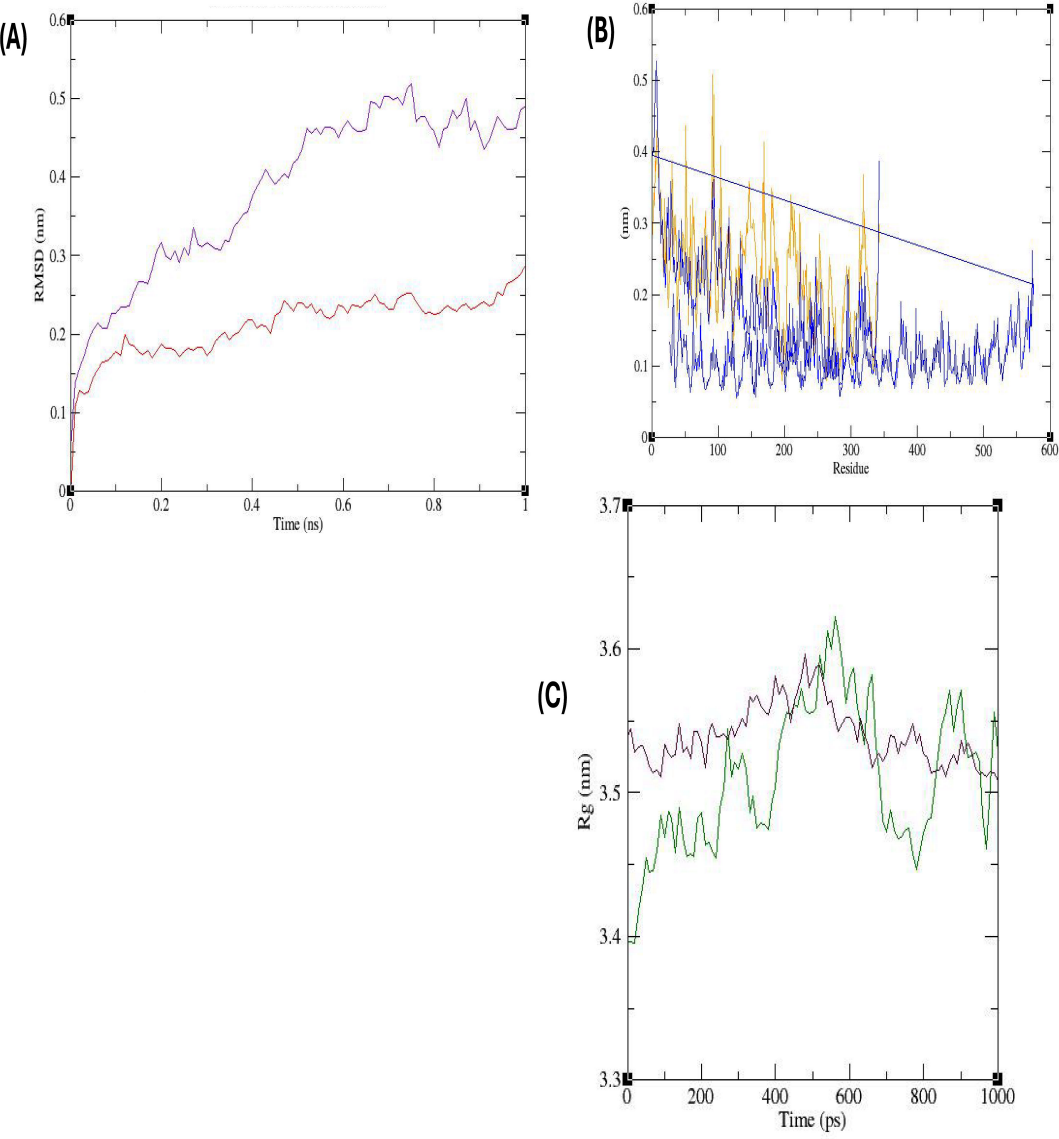
Molecular dynamics simulation conducted on the protein ACA31267.1 receptor-complex, accompanied by a comprehensive evaluation based on RMSD, RMSF, and Rg. (**A**) The RMSD plot provides insights into the stability of the protein-receptor complex. The complex itself is depicted in red color, while the protein without the associated receptor is portrayed in a contrasting violet color; (**B**) RMSF plot, highlighting the flexibility of individual residues within the system. The docked complex is presented in blue, while the protein in isolation, devoid of the receptor, is yellow-orange; (**C**) the Rg, indicating the compactness of the protein structure. The complex structure is illustrated in a deep maroon color, whereas the stand-alone protein is represented in green.

The structural dynamics of the proteins were investigated by analyzing their root mean square deviation (RMSD) values. In the case of the ligand-receptor complex ACA31267.1 with bovine TLR2, the RMSD plot demonstrated stability throughout the entire simulation, with consistently lower RMSD values compared to the single protein. Conversely, the single protein without the receptor exhibited an initial increase in RMSD from 0.1 to 0.5 nm, followed by a relatively stable behavior for the remainder of the simulation (Fig. 7A). The flexibility of individual residues within the proteins was assessed using the root mean square fluctuation (RMSF), where higher values indicate greater flexibility. For the protein-receptor complex ACA31267.1-bovine TLR2 and the single protein, the RMSF plot revealed that the single protein exhibited more pronounced fluctuations, with peaks reaching up to 0.5 nm, whereas the complex structure was more stable (Fig. 7B). The radius of gyration (Rg) was utilized to evaluate the molecular compactness of the proteins, reflecting the distribution of atoms around their axis. In the case of protein ACA31267.1, the Rg value exhibited notable fluctuations, reaching a peak of 3.6 nm. Conversely, the complex protein (ACA31267.1-TLR2) demonstrated a narrower distribution, with a peak lower than that of the single protein (Fig. 7C).

## DISCUSSION

Although vaccination using killed bacteria is a cost-effective intervention aimed at minimizing the incidence of BRD, current *H. somni* vaccines containing killed, planktonic bacteria have only been moderately to poorly effective in protecting cattle from BRD (23–26). This failure may, in part, be attributed to the lifestyle of *H. somni* in the host (particularly in BRD), which is predominantly as a biofilm (5). Most of the *H. somni* biofilm matrix consists of EPS, which is not produced when the bacteria are grown planktonically (12). Other important factors to consider in vaccine design are how the antigens are presented to the host by the bacteria, what type of immune response by the host is required for protection against opportunistic pathogens, and the bacterial lifestyle in the host. Furthermore, within the host, *H. somni* decorates its LOS and biofilm EPS with host-derived sialic acid, which is not present in culture medium (11, 12). *H. somni* must also produce iron binding proteins in the host to sequester iron for growth, but these regulated proteins are not produced in culture medium, which has a rich supply of free iron (13, 27). To begin formulating new vaccine candidates that are relatively simple to prepare and harbor more host-relevant antigens, spontaneously released proteins in *H. somni* OMVs were examined under iron-restricted and iron-rich growth conditions, as well as during biofilm formation. We also identified potential protective antigens for future vaccine consideration through web-based bioinformatics tools. However, one limitation of current bioinformatics tools is the lack of information regarding tertiary gene products (lipids and polysaccharides).

OMVs are small spherical structures derived from the cell envelope of gram-negative bacteria (28, 29) and are commonly produced under diverse growth environments, including planktonic cultures (30), biofilms (31), and within mammalian hosts (32). OMVs that contain transferrin-binding proteins may also induce protective immunity through blocking bacterial acquisition of iron (33, 34). The TonB-dependent receptor TbpA and the surface lipoprotein TbpB are two well-characterized Tbps utilized by bacteria to sequester iron from transferrins in the host. Apart from these two proteins, homologs of TbpA2, which resemble TbpA and other Tbps, have been reported in *Pasteurella multocida* (35) and *H. somni* (36). In this work, TbpA (ACA32419.1; HSM_0750), TbpB (ACA32417.1 HSM_0749), and a TbpA2 homolog (ACA31785.1; HSM_1988) were identified in OMVs of *H. somni* regardless of the availability of iron in the environment but were in greater abundance under EDDHA treatment. By contrast, two homologs of TbpA2 (ACA32614.1; HSM_0931 and ACA32615.1; HSM_0932) were exclusively present in OMVs under EDDHA treatment (Table S1). TbpA2 is present exclusively in gram-negative bacteria that colonize the oral or oropharyngeal mucosa of ruminants (37). Through NCBI BLAST analysis, gram-negative bacteria like *Actinobacillus rossini* and *Campylobacter majalis* express proteins homologous to proteins ACA32614.1 and ACA32651.1, the two unique proteins detected under EDDHA treatment in this study. Five QS-associated proteins were detected under the growth conditions that promoted biofilm formation. Since QS is a bacterial cell-to-cell communication system known to regulate group behaviors, including biofilm development (38–40), it is not surprising that various bacteria possess homologs of the QS-associated proteins found in *H. somni*. More than 10 bacterial species from different genera have a homolog of the QS-associated *H. somni* protein Sec (ACA31843.1). Phase variation can occur in bacterial surface structures and virulence factors, including adhesins (41), iron-acquisition proteins (42), pili (43), and LOS biosynthetic enzymes due to multiple genetic mechanisms (44, 45). The expression of iron-regulated outer membrane proteins, including hemoglobin-binding protein HgbA (HSM_1168), transferrin-binding protein TbpB (HSM_0749), and TonB-dependent lactoferrin and transferrin receptor TbpA2 (HSM_1988), has been reported to be phase variable (36, 42, 46). Therefore, phase variation of TbpB and TbpA2, which were identified in OMVs in *H. somni*, may contribute to the differential regulation of these two TbpA-like proteins.

In agreement with transcriptomic data from previous studies (16, 47–49), the protein profile of the biofilm matrix revealed that *H. somni* can change its metabolic status during biofilm formation at both the transcriptional (16, 50) and translational levels (this work). Three hundred seventy-six proteins were exclusively present in the *H. somni* biofilm matrix, which supports previous studies that about half of the *H. somni* genome is differentially expressed when the bacteria are in a biofilm compared to planktonic growth (16, 50). Thus far, neither OMVs nor any novel biofilm matrix components have been tested for protective efficacy, but such studies are currently underway.

Analysis of cellular processes for a QS signaling network using the KEGG server indicated that *H. somni* possessed six genes homologous to the genes in the *Escherichia coli lsr* (*lux*S-regulated) operon (51, 52), which are responsible for transporting and processing the QS signal molecule AI-2 (52). We have previously reported that RNA expression of *luxS* (HSM_1663), five genes (HSM_1944, 1945, and 1947–1949) of the *lsr* (*luxS*-regulated) operon (annotated based on homologous genes in *E. coli*, including HSM_1944–1949), and two genes (HSM_1942–1943) adjacent to the *lsr* operon were upregulated when *H. somni* was grown as a biofilm (16). In the present study, we identified two proteins corresponding to the above-mentioned genes: HSM_1948 was exclusively identified in the biofilm matrix, whereas HSM_1947 was present during all three tested growth conditions (Table S1). Transcriptomic analysis (16) revealed that the log2 fold change (1.82653) in the gene expression level of HSM_1948 during biofilm growth was nearly double that of HSM_1947 (0.980441). These results suggest that the regulation of individual genes within the *lsr* operon and their corresponding proteins follows a complex and hierarchical pattern.

An immuno-bioinformatic approach was employed to screen a collection of 92 proteins expressed during different *H. somni* growth conditions, particularly conditions that simulate the growth environment in the host. Among these proteins, 19 were identified as outer membrane/extracellular proteins, and two specific proteins (ACA31267.1 and ACA32419.1) were determined to contribute to virulence based on analysis using the VFDB. The VFDB contains the DNA/Protein sequence homology of bacterial pathogens and can be used to identify potential virulence factors in other species by comparative analysis. The presence of signal peptides and transmembrane helices was examined, as surface-exposed proteins typically have signal peptides for secretion to the bacterial outer membrane and possess a minimum number of helices to ensure that a significant portion of the protein remains exposed to the immune system. In addition, the antigenicity scores of these proteins were examined using bioinformatic tools such as VaxiJen, which predicts antigenic profiling of these proteins based on their physicochemical properties. The identified proteins were surface exposed, possessed a signal peptide, and had a good antigenicity score, indicating they may be good vaccine targets. ACA31267.1 was identified as an *H. somni* OmpA outer membrane protein, which has been shown to be expressed on the bacterial surface, is highly conserved in all strains examined, and induces an antibody response (53). OmpA family proteins are commonly found in other bacteria, such as *E. coli* (54), *Klebsiella pneumoniae* (55), and *Acinetobacter baumannii* (56). In *E. coli* and *K. pneumoniae,* OmpA plays an important role in bacterial adhesion to host epithelial cells and evasion of host immune response. OmpA has also been shown to aid in biofilm formation (57). ACA31267.1 was present in OMV preparations from cells grown with or without iron, as well as in biofilm matrix preparations (Fig. 1, black arrow). ACA32419.1 was annotated as a TonB-dependent receptor homolog. Although TonB is a transporter of siderophores and other molecules (58, 59), TonB has not been previously identified as an immuno-protective antigen in *H. somni*. However, *Neisseria gonorrhoeae* can utilize TonB receptors for iron acquisition (60). Therefore, it is reasonable to conclude that blocking the transport of essential components into the bacterium would compromise virulence. Due to their surface exposure and role in bacterial virulence, TonB-dependent receptor and OmpA proteins are logical vaccine candidates. Even if these proteins are present in the current killed vaccines, they may not stimulate a protective immune response due to the process used to kill the bacteria, interference by other antigens, or other factors affecting the accessibility of the antigens to the host while on intact bacteria. Therefore, these proteins may be more effective vaccine candidates in a more concentrated form, such as on OMVs. Moreover, both proteins were found to be non-homologous to bovine proteins, a crucial consideration to avoid potential autoimmunity issues. Similar strategies have been successfully employed in predicting vaccine candidates for other pathogens, such as *Clostridium chauvoei* (61), *Serratia marcescens* (62), and *Mycobacterium avium* (63).

The tertiary structure provides information about a protein’s interaction potential and accessibility to host immune system receptors. The tertiary structure of OmpA- and TonB-dependent receptor was supported using the Ramachandran plot. Notably, the majority of residues (>90%) within both proteins were observed to reside within the permissible region, indicating a favorable conformational arrangement. TLR2 is a component of the host innate immune response and is responsible for recognizing pathogen-associated molecular patterns (PAMPs) that are bacterial proteins. The molecular docking with favorable low binding energy indicates which proteins can act as a PAMP, effectively stimulating the host immune response. As the crystal structure of bovine TLR2 is unavailable, the sequence from UniProt (TLR2- Q95LA9) for homology modeling using Modeller 10.4 was used. In the docking study, both proteins displayed a favorable binding affinity when interacting with TLR2. The protein-ligand complex of ACA31267.1-TLR2 had a much lower binding energy score than that of the ACA32419.1-TLR2 complex, suggesting a stronger and more stable interaction that could potentially contribute to enhanced immunogenicity. Nevertheless, *in vivo* and *in vitro* experiments are required to confirm the protective efficacy of the protein.

Some proteins, such as IbpA, which are known to elicit effective immune responses based on previous experimental vaccine studies (7, 64), were excluded from the virulence prediction in this work due to their absence of signal peptides. Despite not having classical signal peptides and being excluded as potentially protective antigens in this study, the DR2 Fic motif of IbpA (7, 64) and the OmpP1 homologs CBN71110 and CBN71009 in *Haemophilus influenzae* (65) have been demonstrated to effectively elicit favorable immune responses in previous experimental vaccine studies. In fact, two secretion motifs (NPNL and NPNGI) found in a family of secreted proteins (66) are conserved in the IbpA protein (67), indicating that this protein is transported to the bacterial surface by a two-partner secretion system (a subclass of type V secretion system) (68). Thus, another limitation of current bioinformatic programs is that signal peptides/secretion domains in the two-partner secretion system cannot be adequately identified using prediction methods such as the PSORT (69) or SignalP 5.0 (70) servers since they only cover the prediction of Type III secretion and Sec-/Tat-dependent secretion systems. Furthermore, while the SecretomeP server (71) has been developed to predict non-classically secreted proteins, this server cannot process proteins with more than 4,000 amino acids, such as the IbpA protein (4,095 amino acids). Nonetheless, bioinformatics analysis can be used to identify new candidate antigens to be combined to improve vaccine efficacy.

### Conclusions

In summary, transferrin-binding proteins were shown to be present in *H. somni* OMVs, supporting the potential participation of OMVs in iron acquisition. The exclusive presence of TbpA2 homologs in the OMVs under EDDHA treatment reflected differences in TbpBA and TbpA2 homologs regarding their production and transport by OMVs. Many more proteins (primarily in the cytoplasm) were present in the biofilm matrix than in OMV, suggesting an immune response to these proteins may aid in reducing or preventing biofilm formation in the host. For example, striped catfish immunized with biofilm from *Aeromonas hydrophila* demonstrated significantly greater weight gain, specific antibody titers, and survival following lethal challenge with *A. hydrophila* in comparison to fish immunized with planktonic cells (72). Furthermore, Mirzaei et al. (73) have reviewed and discussed the advantages of using staphylococcal biofilms as vaccine antigens. Based on bioinformatics analysis, ACA31267.1 (OmpA) and ACA32419.1 (TonB-dependent receptor) were identified as potential protective antigens. These results may be valuable in the development of future vaccines to prevent BRD and other systemic infections associated with *H. somni*.

## MATERIALS AND METHODS

### Bacteria

*H. somni* strain 2336 is a pathogenic isolate from bovine pneumonia (74) and maintained in 10% skim milk at −80°C. Stock cultures were inoculated onto Columbia blood agar (BD Becton-Dickinson and Company [BD], Franklin Lakes, NJ, USA) and incubated overnight at 37°C in 5% CO_2_.

### Bacterial growth in an iron-sufficient or iron-deficient environment

Iron availability was controlled during *H. somni* growth as described by Ogunnariwo et al. (27) with modifications. *H. somni* colonies from a Columbia blood agar plate were inoculated into brain heart infusion broth (BD) supplemented with 0.5% yeast extract 0.1% Trizma base and(75) to prepare 40 mL of the starter culture with an initial optical density (OD) of 0.8 at 600 nm (OD_600_). The starter culture was diluted 1 to 40 into a 40 mL and a 200 mL culture, which were further supplemented with 0.01% thiamine monophosphate (TMP, Sigma-Aldrich) to obtain BHI-yeast exctract-Tris-TMP (BYTT). The 40 mL starter culture was inoculated with 0.4 mL of 10 mM filter-sterilized ferric nitrate (Sigma-Aldrich) to create an iron-sufficient environment. To generate the iron-deficient environment, 2 mL of filter-sterilized 10 mM EDDHA was added to the 200 mL culture. The cultures were then incubated at 37°C and shaken at 200 rpm until the OD_600_ of the ferric nitrate-treated culture reached 0.8 (the OD_600_ of the EDDHA-treated culture was about 0.25, as EDDHA limited bacterial growth). A representative growth curve of *H. somni* in BYTT supplemented with and without EDDHA is shown in Fig. S2. The treated cultures were centrifuged at 9,000 × *g* for 15 min at 4°C. The supernatant was filter sterilized (0.2 µm filter) and kept at 4°C for the preparation of OMVs.

### Biofilm growth and matrix preparation

*H. somni* biofilms were grown in supplemented Columbia broth to 10^9^ colonyforming units/mL as previously described (12) with modification. The bacterial culture (0.5 mL) was transferred to 50 mL of CTT containing 1% glucose in a 60 mL plastic conical tube, followed by static incubation for 5 days at 37°C for biofilm formation. On the third day of incubation, the top half of the culture was replaced with fresh broth. After incubation, the medium was gently removed, and 1 mL of phosphate-buffered saline (PBS) was added to flush out the biofilm biomass attached to the tube wall with repetitive pipetting. The suspension in the tube was then vortexed for 20 min at maximum speed to homogenize the material (76), and the bacteria were pelleted by centrifugation at 9,000 × *g* for 15 min at 4°C. The supernatant was sterilized by passage through a 0.2 µm filter and kept at −20°C.

### OMV preparation

The OMVs released by *H. somni* grown with ferric nitrate or EDDHA were collected from the culture supernatant (40 or 200 mL) as described (77), with modification. Briefly, the OMVs were collected from the culture supernatant by ultracentrifugation at 170,000 × *g* for 4 h at 4°C (SW 32 Ti rotor; 25 × 89 mm Open-Top Thinwall Ultra-Clear tube; Beckman-Coulter, Brea, CA, USA). The pelleted OMVs were suspended with sterile PBS containing 1× protease inhibitors (Thermo Fisher Scientific, Waltham, MA, USA), filter sterilized, and stored at −20°C.

### Transmission electron microscopy

A portion of the OMVs from bacteria incubated with EDDHA was pelleted by centrifugation, resuspended in a small volume of 2.5% glutaraldehyde, and processed for transmission electron microscopy with a Hitachi HT7800 microscope at the Cold Spring Harbor Core Microscopy Shared Resource Laboratory.

### Electrophoretic analysis

The protein concentration in the samples was determined using the Pierce BCA protein assay (Thermo Fisher Scientific) following the manufacturer’s instructions. Samples containing 20–30 µg of protein were prepared as described (78), resolved by electrophoresis through 4%–12% Bis-Tris gels (Thermo Fisher Scientific), and stained with Page Blue protein staining solution (Thermo Fisher Scientific), as described (79). Proteins from two independent replicates for each of the three different growth conditions (OMVs from an iron-sufficient environment, OMVs from an iron-deficient environment, and biofilm matrix) were analyzed by sodium dodecyl sulfate-polyacrylamide gel electrophoresis. Each lane on the gel (Fig. 1, for example) was split into four sections for proteomic analysis.

### Proteomic analysis

Proteomic analysis was carried out at the Proteomics and Metabolomics Facility at the Biotechnology Resource Center of Cornell University Institute of Biotechnology. Proteins in the gel pieces were reduced, alkylated, in-gel digested, cleaned, and analyzed using an LTQ Orbitrap Velos mass spectrometer (Thermo Fisher Scientific) equipped with an Advion nanomate ESI source (Advion). Tandem mass spectra were processed by the SEQUEST (Xcorr) algorithm (80), and results were searched against the NCBI database of *H. somni* strain 2336 (https://www.ncbi.nlm.nih.gov/nuccore/168825335). Scaffold version 4.8.4 (Proteome Software Inc.) was used to validate the MS/MS-based protein and peptide identifications. Peptide identifications were accepted if they were established at greater than 95.0% probability by the Scaffold Local FDR algorithm. Protein identifications were accepted if they could be established at greater than 99.9% probability and contained at least two identified peptides. Proteins identified in both replicates of each growth condition were selected for further analysis.

### Bioinformatic analysis

#### *In silico* analysis to identify potential protective antigens

The common proteins identified under the three different growth conditions (iron-sufficient, EDDHA-treated, and biofilm matrix) were analyzed using PSORTb version 3.0.3 (https://www.psort.org/psortb/index.html) (81) for cellular localization. The proteins localized to OM/EC with the potential for contributing to virulence were searched for by querying against the entire protein data set of the VFDB (http://www.mgc.ac.cn/VFs/search_VFs.htm) (82) using local blast 2.13.0+. Stringent criteria of a minimum sequence identity of 50% and a query coverage of no less than 70% were employed. The antigenicity of the selected proteins was assessed using the VaxiJen version 2.0 (https://www.ddg-pharmfac.net/vaxijen/VaxiJen/VaxiJen.html) (83) tool at a threshold of ≥0.4, which employs predictive algorithms to determine their potential as antigens. Furthermore, transmembrane helices were evaluated using Vaxign 2 (https://violinet.org/vaxign2) (84). The presence of signal peptides in proteins ACA31267.1 and ACA32419.1 was assessed using SignalP 5.0 (70). To ascertain the degree of resemblance between virulent proteins and the bovine proteome, a meticulous local blastp analysis was employed using version 2.13.0+. The blast output was then subjected to filtration, excluding proteins exhibiting a percent identity score of ≥25 and a percent query coverage of ≥30 within the bovine protein data set (85, 86).

### Molecular docking and simulation

AlphaFold3 (https://alphafoldserver.com/) was used to construct the tertiary structure. The quality of the generated tertiary model was assessed through the Ramachandran plot using the R software, which provides insights into the geometry and stereochemical properties of the refined protein structure. To assess the interaction between proteins ACA31267.1 and ACA32419.1 and bovine immune receptors, an in-depth molecular docking analysis was performed using the standalone HDOCK docking tool (87), which monitors the precise binding of a protein to the bovine TLR2 immune receptor. Subsequently, the obtained docking models were refined using the HADDOCK refinement method. Intramolecular interactions such as hydrogen bonding and the key residues at the interface between protein and receptor were determined using the standalone DIMPLOT program of LigPlot+. The prodigy server was employed to calculate the binding energy resulting from the interaction between the protein and receptor.

The computational framework Gromacs 2023 (https://anaconda.org/conda-forge/gromacs) was used to investigate the structural integrity and energetic optimization of the Protein-TLR2 complex. Through the utilization of pdb2gmx, a precise protein topology file was generated, incorporating the high-fidelity Amber99SB force field. To simulate the complex, the TIP3P water model was selected, ensuring an accurate representation of the solvent environment. The system’s charge neutrality was maintained by the addition of chloride and sodium ions. Subsequently, an energy minimization procedure was carried out, followed by thorough equilibration under isothermal and isobaric conditions at 300 K and 1 bar, respectively. A detailed molecular dynamics simulation, spanning a duration of 1 ns, was conducted to capture the intricate dynamics of the Protein-TLR complex. Various analysis techniques, including RMSD, RMSF, and Rg, were employed to examine the complex’s stability and intermolecular interactions.

## Data Availability

The mass spectrometry proteomics data have been deposited to the ProteomeXchange Consortium via the PRIDE partner repository with the datasetdata set identifier PXD061987. The reviewer can access the datasetdata set by logging into the PRIDE website (https://www.ebi.ac.uk/pride/archive) using the following account details: Username: reviewer_pxd061987@ebi.ac.uk; Password: wrSgVEXI1SZI, and then click "review submission".

## SUPPLEMENTAL MATERIAL

Supplemental Tables and Figures mbio.00644-25-s0001.pdf Fig. S1 and S2 and Tables S1, S2, and S3.

ASM does not own the copyrights to Supplemental Material that may be linked to, or accessed through, an article. The authors have granted ASM a non-exclusive, world-wide license to publish the Supplemental Material files. Please contact the corresponding author directly for reuse.
