# Supplementary material for: Characterization of proteins present in the biofilm matrix and outer membrane vesicles of Histophilus somni during iron-sufficient and iron-restricted growth: identification of potential protective antigens through in silico analyses
Source: mBio. 2025 Apr 17;16(5):e00644-25. doi: 10.1128/mbio.00644-25 (PMC12077179; doi:10.1128/mbio.00644-25)
Supplement: Supplemental Tables and Figures — Fig. S1 and S2 and Tables S1, S2, and S3. [file mbio.00644-25-s0001.pdf]

## Supplemental tables and figures

**Supplemental Table S1. Characterization of the proteins identified in this study by source.**

| Groups [a] | Protein ID | Gene locus tag | Protein Description | MW [kDa] | OMV_Fe | | OMV_EDDHA | | Biofilm | |
|---|---|---|---|---|---|---|---|---|---|---|
| | | | | | Protein abundance [b] | | | | | |
| | | | | | Average | Standard deviation | Average | Standard deviation | Average | Standard deviation |
| Unique proteins | | | | | | | | | | |
| OMV_Fe | ACA30734.1 | HSM_1022 | YadA domain protein | 396.9 | 148238.22 | 167566.68 | | | | |
| OMV_Fe | ACA31054.1 | HSM_0132 | Tol-Pal system TolQ | 25.5 | 499266.87 | 128397.70 | | | | |
| OMV_Fe | ACA31654.1 | HSM_1864 | conserved hypothetical protein | 4.7 | 31433338.59 | 3390451.80 | | | | |
| OMV_Fe | ACA31752.1 | HSM_1958 | ribosomal protein S3 | 25.8 | 1378047.13 | 1117594.82 | | | | |
| OMV_Fe | ACA32015.1 | HSM_0377 | YadA domain protein | 343.1 | 4250509.46 | 5117657.27 | | | | |
| OMV_Fe | ACA32034.1 | HSM_0394 | YadA domain protein | 344.8 | 860322.19 | 1182579.74 | | | | |
| OMV_Fe | ACA32047.1 | HSM_0405 | anti sigma-E protein, RseA | 22.3 | 575105.61 | 40337.69 | | | | |
| OMV_Fe | ACA32075.1 | HSM_0430 | Monosaccharide-transporting ATPase | 34.1 | 251991.03 | No Quan Value [c] | | | | |
| OMV_Fe | B0UVM9.1 | HSM_0168 | 50S ribosomal protein L19 | 13.2 | 3655138.16 | 1963866.48 | | | | |
| OMV_Fe | B0UX32.1 | HSM_1971 | 50S ribosomal protein L30 | 6.8 | 7120264.55 | 3679470.87 | | | | |
| OMV_EDDHA | ACA30786.1 | HSM_1071 | penicillin-binding protein, 1A family | 93.5 | | | No Quan Value | No Quan Value | | |
| OMV_EDDHA | ACA31186.1 | HSM_1440 | nuclease (SNase domain protein) | 19.8 | | | 124321.55 | No Quan Value | | |
| OMV_EDDHA | ACA31510.1 | HSM_1732 | protein of unknown function DUF305 | 27.1 | | | 227480.05 | No Quan Value | | |
| OMV_EDDHA | ACA32586.1 | HSM_0906 | conserved hypothetical protein | 33.1 | | | 893460.31 | 1082275.44 | | |
| OMV_EDDHA | ACA32592.1 | HSM_0911 | hypothetical protein HSM_0911 | 51.4 | | | 444151.27 | 399819.37 | | |
| OMV_EDDHA | ACA32614.1 | HSM_0931 | TonB-dependent receptor | 68.1 | | | 891417.65 | 41000.97 | | |
| OMV_EDDHA | ACA32615.1 | HSM_0932 | TonB-dependent receptor plug | 24.6 | | | 615323.16 | 283016.11 | | |
| Biofilm | ACA30710.1 | HSM_1000 | TM2 domain containing protein | 14 | | | | | 230404.36 | 38383.21 |
| Biofilm | ACA30711.1 | HSM_1001 | exoribonuclease II | 75.8 | | | | | No Quan Value | No Quan Value |
| Biofilm | ACA30712.1 | HSM_1002 | short-chain dehydrogenase/reductase SDR | 28 | | | | | 5867834.99 | 696697.98 |
| Biofilm | ACA30713.1 | HSM_1003 | protein of unknown function DUF34 | 28 | | | | | 1078499.64 | 191490.80 |
| Biofilm | ACA30715.1 | HSM_1005 | Beta-ketoacyl synthase | 42.8 | | | | | 319059477.21 | 122968545.09 |

| | | | | | | | | | | |
|---|---|---|---|---|---|---|---|---|---|---|
| Biofilm | ACA30719.1 | HSM_1009 | Phosphotransferase system, phosphocarrier protein HPr | 9 | | | | | 33480355.57 | 18538846.28 |
| Biofilm | ACA30721.1 | HSM_1010 | phosphoenolpyruvate-protein phosphotransferase | 63.6 | | | | | 23457467.91 | 6076922.53 |
| Biofilm | ACA30722.1 | HSM_1011 | PTS system, glucose subfamily, IIA subunit | 18.1 | | | | | 3452077.83 | 2159489.44 |
| Biofilm | ACA30733.1 | HSM_1021 | pyruvate kinase | 51.5 | | | | | 181589298.38 | 12383536.08 |
| Biofilm | ACA30737.1 | HSM_1025 | alkylhydroperoxidase like protein, AhpD family | 12 | | | | | 58801404.10 | 4105417.90 |
| Biofilm | ACA30740.1 | HSM_1028 | thioredoxin reductase | 34.2 | | | | | 16323287.76 | 3129269.15 |
| Biofilm | ACA30757.1 | HSM_1043 | histidine triad (HIT) protein | 12.9 | | | | | 3241956.73 | 1935520.93 |
| Biofilm | ACA30762.1 | HSM_1048 | single-strand binding protein | 17.4 | | | | | 2961889.00 | 117532.49 |
| Biofilm | ACA30763.1 | HSM_1049 | excinuclease ABC, A subunit | 104.2 | | | | | 1725900.80 | 376721.56 |
| Biofilm | ACA30774.1 | HSM_1059 | UspA domain protein | 15.7 | | | | | 2089798.11 | 1246452.51 |
| Biofilm | ACA30777.1 | HSM_1062 | Phosphomannomutase | 50.4 | | | | | 44888387.20 | 7918743.69 |
| Biofilm | ACA30778.1 | HSM_1063 | UTP-glucose-1-phosphate uridylyltransferase | 32.3 | | | | | 619749.60 | 450756.48 |
| Biofilm | ACA30781.1 | HSM_1066 | Shikimate kinase | 19.8 | | | | | 422682.95 | 450704.38 |
| Biofilm | ACA30788.1 | HSM_1073 | glutathione-disulfide reductase | 49.5 | | | | | 291868096.20 | 65414278.94 |
| Biofilm | ACA30789.1 | HSM_1074 | RNA chaperone Hfq | 10.9 | | | | | 5005573.79 | 2358414.38 |
| Biofilm | ACA30792.1 | HSM_1077 | fumarate hydratase, class II | 50.6 | | | | | 811756191.82 | 136060601.64 |
| Biofilm | ACA30795.1 | HSM_0108 | galactose-1-phosphate uridylyltransferase | 40.7 | | | | | 1589124.66 | 258447.55 |
| Biofilm | ACA30806.1 | HSM_0109 | galactokinase | 43 | | | | | 3490797.89 | 583931.46 |
| Biofilm | ACA30810.1 | HSM_1093 | peptidase M24 | 50.7 | | | | | 68981295.07 | 14003073.54 |
| Biofilm | ACA30818.1 | HSM_0110 | galactose mutarotase | 39.3 | | | | | 3415502.96 | 1445424.08 |
| Biofilm | ACA30822.1 | HSM_1103 | argininosuccinate lyase | 51.4 | | | | | 14406338.28 | 5255714.27 |
| Biofilm | ACA30836.1 | HSM_1116 | translation initiation factor IF-1 | 8.3 | | | | | 430148.32 | 362814.58 |
| Biofilm | ACA30841.1 | HSM_1120 | protein of unknown function DUF224 cysteine-rich region domain protein | 26.7 | | | | | 1096652.80 | 359279.53 |
| Biofilm | ACA30842.1 | HSM_1121 | iron-sulfur cluster binding protein | 52.1 | | | | | 5765631.30 | 2380717.02 |
| Biofilm | ACA30843.1 | HSM_1122 | protein of unknown function DUF162 | 25.9 | | | | | 412259.20 | 320846.83 |
| Biofilm | ACA30866.1 | HSM_1143 | addiction module toxin, Txe/YoeB family | 10.2 | | | | | 717537.07 | 473207.49 |
| Biofilm | ACA30878.1 | HSM_1154 | hypothetical protein HSM_1154 | 13.4 | | | | | 6107241.85 | 2260004.46 |
| Biofilm | ACA30918.1 | HSM_1196 | dihydropteroate synthase | 30.3 | | | | | 869525.34 | 1034329.79 |

| | | | | | | | | | | |
|---|---|---|---|---|---|---|---|---|---|---|
| Biofilm | ACA30947.1 | HSM_1221 | Glutathione S-transferase domain | 24.3 | | | | | 6749744.71 | 2721783.21 |
| Biofilm | ACA30960.1 | HSM_1233 | SMP-30/Gluconolaconase/LRE domain protein | 32.8 | | | | | 336537.19 | No Quan Value |
| Biofilm | ACA30964.1 | HSM_1237 | carbohydrate kinase FGGY | 53.8 | | | | | 224554.17 | 63818.97 |
| Biofilm | ACA30965.1 | HSM_1238 | 3-dehydro-L-gulonate-6-phosphate decarboxylase | 23.7 | | | | | 157571.20 | 4849.26 |
| Biofilm | ACA30966.1 | HSM_1239 | putative hexulose-6-phosphate isomerase | 33.4 | | | | | 305467.76 | 267410.42 |
| Biofilm | ACA30968.1 | HSM_1240 | L-ribulose-5-phosphate 4-epimerase | 26 | | | | | 13365552.01 | 6127131.60 |
| Biofilm | ACA30972.1 | HSM_1244 | Cof-like hydrolase | 30.4 | | | | | 45787.92 | No Quan Value |
| Biofilm | ACA30973.1 | HSM_1245 | nitroreductase | 25.1 | | | | | 1154690.44 | 41661.31 |
| Biofilm | ACA30980.1 | HSM_1251 | D-lactate dehydrogenase | 64.1 | | | | | 9665404.74 | 3037645.89 |
| Biofilm | ACA30981.1 | HSM_1252 | 2,3,4,5-tetrahydropyridine-2,6-dicarboxylate N-succinyltransferase | 29.6 | | | | | 67748711.23 | 16513039.97 |
| Biofilm | ACA30982.1 | HSM_1253 | formyltetrahydrofolate deformylase | 31.8 | | | | | 6083347.87 | 2320774.54 |
| Biofilm | ACA30986.1 | HSM_1257 | YadA domain protein | 484.1 | | | | | 1978699.34 | 1609985.66 |
| Biofilm | ACA30987.1 | HSM_1258 | Oligopeptidase A | 77.9 | | | | | 262721948.05 | 72547445.21 |
| Biofilm | ACA30996.1 | HSM_1268 | dihydroorotate dehydrogenase | 35.5 | | | | | 4769539.52 | 1642560.25 |
| Biofilm | ACA30999.1 | HSM_1270 | tryptophanase | 53 | | | | | 590417426.23 | 51933456.37 |
| Biofilm | ACA31000.1 | HSM_1271 | phosphate acetyltransferase | 77.1 | | | | | 5077520.08 | 2514643.85 |
| Biofilm | ACA31001.1 | HSM_1272 | acetate kinase | 43.3 | | | | | 51391093.70 | 8004547.78 |
| Biofilm | ACA31005.1 | HSM_1276 | NapC/NirT cytochrome c domain protein | 41.7 | | | | | 431637.71 | 51683.70 |
| Biofilm | ACA31011.1 | HSM_1281 | riboflavin synthase, alpha subunit | 22.2 | | | | | 1371350.64 | 732428.92 |
| Biofilm | ACA31028.1 | HSM_1297 | methionine aminopeptidase, type I | 30.5 | | | | | 2876749.90 | 1593039.92 |
| Biofilm | ACA31031.1 | HSM_0013 | acetolactate synthase, large subunit, biosynthetic type | 60.1 | | | | | 591161.58 | 85204.62 |
| Biofilm | ACA31038.1 | HSM_1305 | exodeoxyribonuclease III | 31.4 | | | | | 296229.95 | 63997.12 |
| Biofilm | ACA31043.1 | HSM_0131 | Pol-Pal system-associated acyl-CoA thioesterase | 16.1 | | | | | 7116436.69 | 2637598.83 |
| Biofilm | ACA31053.1 | HSM_1319 | threonyl-tRNA synthetase | 74 | | | | | 2616145.46 | 1835437.01 |
| Biofilm | ACA31060.1 | HSM_1325 | conserved hypothetical protein 730 | 51.7 | | | | | 28470810.37 | 7270212.57 |
| Biofilm | ACA31072.1 | HSM_1336 | phenylalanyl-tRNA synthetase, beta subunit | 88 | | | | | 2720586.77 | 2942631.22 |
| Biofilm | ACA31081.1 | HSM_1346 | conserved hypothetical protein | 42 | | | | | 689935.44 | 10340.12 |
| Biofilm | ACA31086.1 | HSM_1350 | Exodeoxyribonuclease I | 55.7 | | | | | 917214.06 | 573888.80 |

| | | | | | | | | | | |
|---|---|---|---|---|---|---|---|---|---|---|
| Biofilm | ACA31094.1 | HSM_1358 | UspA domain protein | 34.9 | | | | | 289409.42 | 367234.92 |
| Biofilm | ACA31098.1 | HSM_1361 | putative Usg-1 protein | 35.2 | | | | | 265671.48 | 205610.07 |
| Biofilm | ACA31100.1 | HSM_1363 | aminopeptidase N | 100.4 | | | | | 199649317.71 | 37586386.13 |
| Biofilm | ACA31102.1 | HSM_1365 | glycogen/starch synthase, ADP-glucose type | 54.1 | | | | | 860130.67 | No Quan Value |
| Biofilm | ACA31111.1 | HSM_1373 | glutaminyl-tRNA synthetase | 63.8 | | | | | 13257747.27 | 2699002.01 |
| Biofilm | ACA31124.1 | HSM_1385 | beta-hydroxyacyl-(acyl-carrier-protein) dehydratase FabA | 15.3 | | | | | 2799535.60 | 1279266.81 |
| Biofilm | ACA31126.1 | HSM_1387 | nitroreductase | 20.7 | | | | | 341721.30 | 224537.79 |
| Biofilm | ACA31132.1 | HSM_1392 | DNA topoisomerase I | 98.5 | | | | | 5579700.53 | 2391584.38 |
| Biofilm | ACA31136.1 | HSM_1396 | KpsF/GutQ family protein | 33.8 | | | | | 631343.14 | 6625.43 |
| Biofilm | ACA31137.1 | HSM_1397 | 3-deoxy-D-manno-octulosonate 8-phosphate phosphatase, YrbI family | 19.8 | | | | | 1294465.10 | 319420.80 |
| Biofilm | ACA31138.1 | HSM_1398 | Ferroxidase | 18.8 | | | | | 73351887.55 | 2067841.30 |
| Biofilm | ACA31139.1 | HSM_1399 | Ferroxidase | 19 | | | | | 21556046.29 | 668486.11 |
| Biofilm | ACA31140.1 | HSM_0014 | acetohydroxy acid synthase II, small subunit | 8.4 | | | | | 249213.75 | 156901.05 |
| Biofilm | ACA31146.1 | HSM_1404 | 2,4-diaminobutyrate 4-transaminase | 49.3 | | | | | 22812073.31 | 10849729.92 |
| Biofilm | ACA31159.1 | HSM_1416 | phospho-2-dehydro-3-deoxyheptonate aldolase | 39.6 | | | | | 8974196.79 | 3499624.41 |
| Biofilm | ACA31164.1 | HSM_1420 | D-isomer specific 2-hydroxyacid dehydrogenase NAD-binding | 34.5 | | | | | 39635929.06 | 13744338.63 |
| Biofilm | ACA31176.1 | HSM_1431 | succinyl-CoA synthetase, alpha subunit | 30.3 | | | | | 1632085.11 | 702627.01 |
| Biofilm | ACA31178.1 | HSM_1433 | 2-oxoglutarate dehydrogenase, E2 subunit, dihydrolipoamide succinyltransferase | 44.7 | | | | | 1739573.35 | 793488.00 |
| Biofilm | ACA31179.1 | HSM_1434 | 2-oxoglutarate dehydrogenase, E1 subunit | 105.9 | | | | | 1021356.27 | 879653.73 |
| Biofilm | ACA31180.1 | HSM_1435 | citrate synthase I | 48.2 | | | | | 1975537.52 | 392696.00 |
| Biofilm | ACA31181.1 | HSM_1436 | Ribonucleoside-diphosphate reductase | 43.3 | | | | | 13983879.04 | 2108540.44 |
| Biofilm | ACA31184.1 | HSM_1439 | putative deoxyguanosinetriphosphate triphosphohydrolase | 52.4 | | | | | 6039102.09 | 2984481.88 |
| Biofilm | ACA31192.1 | HSM_1446 | addiction module toxin, RelE/StbE family | 10.5 | | | | | 2637004.97 | 291045.35 |
| Biofilm | ACA31194.1 | HSM_1448 | virulence-associated protein D (VapD) conserved region | 10.6 | | | | | 47711807.44 | 18223198.95 |
| Biofilm | ACA31201.1 | HSM_1454 | ribosomal protein S1 | 59.8 | | | | | 2350463.96 | 892353.81 |

| | | | | | | | | | | |
|---|---|---|---|---|---|---|---|---|---|---|
| Biofilm | ACA31212.1 | HSM_1464 | ribosome recycling factor | 20.9 | | | | | 21562002.17 | 4872269.49 |
| Biofilm | ACA31213.1 | HSM_1465 | uridylate kinase | 25.7 | | | | | 787297.70 | 220337.14 |
| Biofilm | ACA31223.1 | HSM_1474 | ribose-phosphate pyrophosphokinase | 34.1 | | | | | 30157950.34 | 14163496.37 |
| Biofilm | ACA31227.1 | HSM_1478 | transcription elongation factor GreA | 17.6 | | | | | 874546.07 | 968617.49 |
| Biofilm | ACA31240.1 | HSM_0149 | peptidase U62 modulator of DNA gyrase | 52.1 | | | | | 21948591.59 | 4954715.08 |
| Biofilm | ACA31254.1 | HSM_1501 | transcriptional regulator, TraR/DksA family | 16.8 | | | | | 2163982.78 | 1312425.70 |
| Biofilm | ACA31257.1 | HSM_1504 | N-acetylglucosamine-6-phosphate deacetylase | 41.7 | | | | | 6881250.81 | 4135603.63 |
| Biofilm | ACA31259.1 | HSM_1506 | molybdopterin biosynthesis MoaE protein | 17.2 | | | | | 872020.56 | 133057.28 |
| Biofilm | ACA31272.1 | HSM_1518 | ROK family protein | 33.5 | | | | | 4521598.70 | 76096.41 |
| Biofilm | ACA31307.1 | HSM_0155 | FeS cluster assembly scaffold IscU | 13.6 | | | | | 85824.92 | 79625.45 |
| Biofilm | ACA31318.1 | HSM_0156 | iron-sulfur cluster assembly protein IscA | 11.7 | | | | | 244532.29 | 122291.90 |
| Biofilm | ACA31334.1 | HSM_1574 | spermidine/putrescine ABC transporter ATPase subunit | 42.4 | | | | | 2963975.76 | 2504257.61 |
| Biofilm | ACA31341.1 | HSM_1580 | DNA gyrase, A subunit | 97.9 | | | | | 7559479.21 | 9441507.41 |
| Biofilm | ACA31345.1 | HSM_1584 | UDP-N-acetylglucosamine 1-carboxyvinyltransferase | 45.7 | | | | | 374482.68 | 112479.51 |
| Biofilm | ACA31358.1 | HSM_1596 | methionyl-tRNA synthetase | 77.4 | | | | | 22043251.26 | 9675811.06 |
| Biofilm | ACA31363.1 | HSM_0160 | glutamate--cysteine ligase, putative/amino acid ligase, putative | 85.8 | | | | | 763890.75 | No Quan Value |
| Biofilm | ACA31365.1 | HSM_1601 | NAD(P)H dehydrogenase (quinone) | 23.6 | | | | | 104505.16 | 13866.16 |
| Biofilm | ACA31368.1 | HSM_1604 | 3-dehydroquinate dehydratase, type II | 17.2 | | | | | 10252905.85 | 2851570.62 |
| Biofilm | ACA31369.1 | HSM_1605 | acetyl-CoA carboxylase, biotin carboxyl carrier protein | 16.6 | | | | | 8717712.00 | 4739794.31 |
| Biofilm | ACA31377.1 | HSM_1612 | naphthoate synthase | 31.7 | | | | | 646880.95 | 141935.99 |
| Biofilm | ACA31424.1 | HSM_1655 | ubiquinone/menaquinone biosynthesis methyltransferase | 29.3 | | | | | 63037.89 | No Quan Value |
| Biofilm | ACA31427.1 | HSM_1658 | twin-arginine translocation protein, TatA/E family subunit | 8.2 | | | | | 4556986.64 | 262400.18 |
| Biofilm | ACA31431.1 | HSM_1661 | Porphobilinogen synthase | 38.2 | | | | | 10834738.80 | 4177797.83 |
| Biofilm | ACA31438.1 | HSM_1668 | CTP synthase | 59.8 | | | | | 1501854.73 | 1975606.76 |
| Biofilm | ACA31464.1 | HSM_1691 | conserved hypothetical protein | 72.5 | | | | | 540992.76 | 600542.34 |
| Biofilm | ACA31496.1 | HSM_0172 | phospholipase D/Transphosphatidylase | 53.4 | | | | | 3415843.06 | 1703408.04 |
| Biofilm | ACA31529.1 | HSM_0175 | PfkB domain protein | 70.6 | | | | | 1212118.13 | No Quan Value |

| | | | | | | | | | | |
|---|---|---|---|---|---|---|---|---|---|---|
| Biofilm | ACA31535.1 | HSM_1755 | Leucyl aminopeptidase | 54.1 | | | | | 43345707.38 | 12082497.52 |
| Biofilm | ACA31550.1 | HSM_1769 | lactoylglutathione lyase | 15.2 | | | | | 20783397.56 | 7954652.18 |
| Biofilm | ACA31552.1 | HSM_1770 | ribonuclease T | 24.2 | | | | | 577572.10 | 161545.75 |
| Biofilm | ACA31559.1 | HSM_1777 | branched-chain amino acid aminotransferase | 37.6 | | | | | 5981248.16 | 1859237.97 |
| Biofilm | ACA31560.1 | HSM_1778 | alkyl hydroperoxide reductase/ Thiol specific antioxidant/ Mal allergen | 18 | | | | | 412111.93 | 152910.44 |
| Biofilm | ACA31561.1 | HSM_1779 | dihydrodipicolinate synthase | 31.7 | | | | | 58020333.67 | 24419836.31 |
| Biofilm | ACA31572.1 | HSM_1789 | protein of unknown function DUF328 | 29.5 | | | | | 1561212.54 | 322551.34 |
| Biofilm | ACA31573.1 | HSM_0179 | protein of unknown function DUF413 | 12.6 | | | | | 2263129.84 | 751317.15 |
| Biofilm | ACA31589.1 | HSM_1803 | isocitrate dehydrogenase, NADP-dependent | 80.7 | | | | | 32349298.67 | 7376722.39 |
| Biofilm | ACA31590.1 | HSM_1804 | ATP-dependent carboxylate-amine ligase domain protein ATP-grasp | 45.5 | | | | | 4696312.88 | 914682.40 |
| Biofilm | ACA31600.1 | HSM_1813 | glucose-6-phosphate 1-dehydrogenase | 59.3 | | | | | 18349193.23 | 2462515.10 |
| Biofilm | ACA31601.1 | HSM_1814 | 6-phosphogluconolactonase | 26.5 | | | | | 3996573.16 | 956538.94 |
| Biofilm | ACA31603.1 | HSM_1816 | 6-phosphogluconate dehydrogenase, decarboxylating | 53.3 | | | | | 14490459.52 | 9086741.58 |
| Biofilm | ACA31605.1 | HSM_1818 | YfbU family protein | 19.1 | | | | | 16446343.36 | 4174609.96 |
| Biofilm | ACA31630.1 | HSM_1842 | NADH:ubiquinone oxidoreductase, subunit F | 45.6 | | | | | 314997.75 | 51330.65 |
| Biofilm | ACA31638.1 | HSM_0185 | Haemophilus-specific protein, uncharacterized | 9.9 | | | | | 4996494.36 | 484556.04 |
| Biofilm | ACA31640.1 | HSM_1851 | ATP synthase F1, gamma subunit | 31.9 | | | | | 1702088.18 | 603907.93 |
| Biofilm | ACA31641.1 | HSM_1852 | ATP synthase F1, alpha subunit | 55.8 | | | | | 34734333.47 | 10799169.81 |
| Biofilm | ACA31655.1 | HSM_1865 | diaminopimelate decarboxylase | 46.5 | | | | | 433399.24 | 290990.09 |
| Biofilm | ACA31681.1 | HSM_1893 | Nicotinamide phosphoribosyltransferase | 52.3 | | | | | 3788677.96 | 826049.65 |
| Biofilm | ACA31682.1 | HSM_1894 | Xanthine phosphoribosyltransferase | 17.2 | | | | | 11427816.26 | 3151849.38 |
| Biofilm | ACA31683.1 | HSM_1895 | aminoacyl-histidine dipeptidase | 53.6 | | | | | 58756839.79 | 17332993.76 |
| Biofilm | ACA31703.1 | HSM_1913 | GntR domain protein | 29 | | | | | 82382.19 | 14785.54 |
| Biofilm | ACA31719.1 | HSM_1928 | glutaredoxin-family domain protein | 26.5 | | | | | 3298100.49 | 636734.35 |
| Biofilm | ACA31725.1 | HSM_1933 | methionyl-tRNA formyltransferase | 34.9 | | | | | 63747.06 | 16688.05 |
| Biofilm | ACA31741.1 | HSM_1948 | deoxyribose-phosphate aldolase/phospho-2-dehydro-3-deoxyheptonate aldolase | 31.8 | | | | | 5138920.88 | 2345245.18 |

| | | | | | | | | | | |
|---|---|---|---|---|---|---|---|---|---|---|
| Biofilm | ACA31745.1 | HSM_1951 | ribosomal protein S10 | 11.8 | | | | | 274241.17 | 48230.41 |
| Biofilm | ACA31746.1 | HSM_1952 | ribosomal protein L3 | 22.5 | | | | | 384479.46 | 61900.92 |
| Biofilm | ACA31748.1 | HSM_1954 | Ribosomal protein L25/L23 | 11.3 | | | | | 1148098.11 | 80967.62 |
| Biofilm | ACA31774.1 | HSM_1978 | DNA-directed RNA polymerase, alpha subunit | 36.6 | | | | | 11583946.09 | 4039237.87 |
| Biofilm | ACA31780.1 | HSM_1983 | 3,4-dihydroxy-2-butanone 4-phosphate synthase | 23.2 | | | | | 266333.94 | 60245.70 |
| Biofilm | ACA31790.1 | HSM_1992 | 3-oxoacyl-(acyl-carrier-protein) reductase, putative | 26.1 | | | | | 15827326.40 | 3984750.28 |
| Biofilm | ACA31791.1 | HSM_1993 | Beta-ketoacyl synthase | 45.3 | | | | | 665293.36 | 525534.24 |
| Biofilm | ACA31795.1 | HSM_1997 | phosphopantetheine-binding | 9.4 | | | | | 2031764.81 | 865162.78 |
| Biofilm | ACA31796.1 | HSM_1998 | putative 3-hydroxymyristoyl/3-hydroxydecanoyl-(acyl-carrier-protein) dehydratase | 14 | | | | | 417148.87 | 34775.90 |
| Biofilm | ACA31798.1 | HSM_0002 | DNA polymerase III, beta subunit | 41.3 | | | | | 2530332.50 | 1006815.37 |
| Biofilm | ACA31805.1 | HSM_2004 | conserved thioesterase | 17.5 | | | | | 5954114.58 | 869388.67 |
| Biofilm | ACA31808.1 | HSM_2007 | D-isomer specific 2-hydroxyacid dehydrogenase NAD-binding | 45.1 | | | | | 446255293.92 | 85808947.41 |
| Biofilm | ACA31809.1 | HSM_2008 | ribose 5-phosphate isomerase | 23.2 | | | | | 473892.04 | 426686.67 |
| Biofilm | ACA31814.1 | HSM_2012 | putative endoribonuclease L-PSP | 13.9 | | | | | 64770953.02 | 16601265.04 |
| Biofilm | ACA31818.1 | HSM_2016 | Ferritin Dps family protein | 18.1 | | | | | 126012830.88 | 19929801.40 |
| Biofilm | ACA31829.1 | HSM_0207 | NAD(P)H dehydrogenase (quinone) | 20.5 | | | | | 1089982.72 | 130348.93 |
| Biofilm | ACA31839.1 | HSM_0216 | trigger factor | 48.5 | | | | | 48321150.24 | 22266584.32 |
| Biofilm | ACA31843.1 | HSM_0022 | protein-export protein SecB | 19.5 | | | | | 11588698.40 | 5052690.62 |
| Biofilm | ACA31880.1 | HSM_0253 | Argininosuccinate synthase | 49.6 | | | | | 32428148.50 | 2254624.58 |
| Biofilm | ACA31883.1 | HSM_0256 | acyl-(acyl-carrier-protein)--UDP-N-acetylglucosamine O-acyltransferase | 28.4 | | | | | 207206.22 | No Quan Value |
| Biofilm | ACA31886.1 | HSM_0259 | molybdenum cofactor synthesis domain | 21.3 | | | | | 2192933.07 | 216297.06 |
| Biofilm | ACA31888.1 | HSM_0260 | nitrogen regulatory protein P-II | 12.8 | | | | | 1363186.27 | 944917.85 |
| Biofilm | ACA31893.1 | HSM_0265 | methylglyoxal synthase | 17.2 | | | | | 6846222.84 | 419264.76 |
| Biofilm | ACA31906.1 | HSM_0277 | Cystathionine gamma-synthase | 41 | | | | | 10844152.32 | 1856378.54 |
| Biofilm | ACA31907.1 | HSM_0278 | thioredoxin | 11.8 | | | | | 32613195.77 | 8704718.88 |
| Biofilm | ACA31913.1 | HSM_0283 | Glutamate dehydrogenase (NADP(+)) | 48.6 | | | | | 23213882.09 | 3427431.46 |
| Biofilm | ACA31916.1 | HSM_0286 | PfkB domain protein | 38.3 | | | | | 8496013.39 | 3882534.28 |

| | | | | | | | | | | |
|---|---|---|---|---|---|---|---|---|---|---|
| Biofilm | ACA31917.1 | HSM_0287 | Adenylosuccinate synthase | 47.1 | | | | | 3904186.85 | 3490470.08 |
| Biofilm | ACA31918.1 | HSM_0288 | peptidase U62 modulator of DNA gyrase | 48.8 | | | | | 12013763.83 | 4512410.10 |
| Biofilm | ACA31932.1 | HSM_0030 | ribosomal protein L32 | 6.4 | | | | | 1341113.16 | 784677.51 |
| Biofilm | ACA31935.1 | HSM_0302 | queuine tRNA-ribosyltransferase | 43.2 | | | | | 5240973.12 | 2866186.38 |
| Biofilm | ACA31936.1 | HSM_0303 | preprotein translocase, YajC subunit | 11 | | | | | 521223.64 | 185485.36 |
| Biofilm | ACA31944.1 | HSM_0310 | tyrosyl-tRNA synthetase | 44.3 | | | | | 15337021.61 | 14436505.75 |
| Biofilm | ACA31949.1 | HSM_0317 | peptidylprolyl isomerase FKBP-type | 22.7 | | | | | 560379.04 | 302128.78 |
| Biofilm | ACA31953.1 | HSM_0320 | dihydroneopterin aldolase | 14 | | | | | 2076979.28 | 784327.45 |
| Biofilm | ACA31958.1 | HSM_0325 | YicC domain protein | 33.5 | | | | | 8254485.14 | 2063333.76 |
| Biofilm | ACA31960.1 | HSM_0327 | heat shock protein DnaJ domain protein | 32.8 | | | | | 176350.22 | 128806.67 |
| Biofilm | ACA31962.1 | HSM_0329 | translation elongation factor P | 20.6 | | | | | 3621373.86 | 992306.46 |
| Biofilm | ACA31963.1 | HSM_0033 | malonyl CoA-acyl carrier protein transacylase | 33.7 | | | | | 609852.39 | 22100.89 |
| Biofilm | ACA31982.1 | HSM_0347 | exodeoxyribonuclease VII, large subunit | 50.5 | | | | | 99396.68 | 18804.81 |
| Biofilm | ACA31983.1 | HSM_0348 | alanine racemase domain protein | 27.9 | | | | | 445788.70 | 14410.45 |
| Biofilm | ACA31984.1 | HSM_0349 | aspartate kinase | 88 | | | | | 18956452.98 | 7377525.27 |
| Biofilm | ACA31987.1 | HSM_0351 | threonine synthase | 46.9 | | | | | 4905100.52 | 2041506.38 |
| Biofilm | ACA31988.1 | HSM_0352 | ferric uptake regulator, Fur family | 17 | | | | | 4600926.31 | 1865304.65 |
| Biofilm | ACA31989.1 | HSM_0353 | flavodoxin | 19.7 | | | | | 8510807.72 | 3620616.97 |
| Biofilm | ACA31994.1 | HSM_0358 | Chorismate synthase | 39 | | | | | 606017.24 | 231692.30 |
| Biofilm | ACA31996.1 | HSM_0036 | ribosomal protein L11 | 14.9 | | | | | 1696570.90 | 404855.74 |
| Biofilm | ACA31999.1 | HSM_0362 | adenine phosphoribosyltransferase | 19.6 | | | | | 618985.27 | 159520.49 |
| Biofilm | ACA32016.1 | HSM_0378 | peptidylprolyl isomerase FKBP-type | 21.2 | | | | | 6976829.32 | 1850471.91 |
| Biofilm | ACA32017.1 | HSM_0379 | protein of unknown function DUF1260 | 15.6 | | | | | 1398884.09 | 1304003.24 |
| Biofilm | ACA32019.1 | HSM_0380 | RNA methyltransferase, TrmH family, group 2 | 18.5 | | | | | 811613.41 | 664365.86 |
| Biofilm | ACA32020.1 | HSM_0381 | Phosphoenolpyruvate carboxykinase (ATP) | 59 | | | | | 1521365003.03 | 119839799.54 |
| Biofilm | ACA32022.1 | HSM_0383 | phosphoribosylamine--glycine ligase | 46.4 | | | | | 2492931.48 | 865352.22 |
| Biofilm | ACA32029.1 | HSM_0039 | ribosomal protein L7/L12 | 12.5 | | | | | 57848505.61 | 39623440.82 |
| Biofilm | ACA32035.1 | HSM_0395 | tryptophan halogenase | 46.1 | | | | | 8202496.26 | 3589314.91 |
| Biofilm | ACA32037.1 | HSM_0397 | ADP-L-glycero-D-manno-heptose-6-epimerase | 34.8 | | | | | 7499609.66 | 634303.57 |
| Biofilm | ACA32041.1 | HSM_0040 | transcription termination factor Rho | 47 | | | | | 447184.83 | 419656.25 |

| | | | | | | | | | | | |
|---|---|---|---|---|---|---|---|---|---|---|---|
| Biofilm | ACA32050.1 | HSM_0408 | 2-dehydro-3-deoxyphosphogluconate aldolase/4-hydroxy-2-oxoglutarate aldolase | 23.1 | | | | | | 9390928.84 | 3057915.50 |
| Biofilm | ACA32054.1 | HSM_0411 | glycoside hydrolase family 3 domain protein | 65.3 | | | | | | 22933342.10 | 2163012.12 |
| Biofilm | ACA32055.1 | HSM_0412 | conserved hypothetical protein | 30.8 | | | | | | 357845.27 | 105704.72 |
| Biofilm | ACA32056.1 | HSM_0413 | short-chain dehydrogenase/reductase SDR | 30.1 | | | | | | 7628313.23 | 3337844.54 |
| Biofilm | ACA32058.1 | HSM_0415 | mannonate dehydratase | 44.9 | | | | | | 57525984.66 | 3373036.11 |
| Biofilm | ACA32063.1 | HSM_0042 | Ribulose-phosphate 3-epimerase | 25.1 | | | | | | 7511154.28 | 4509602.42 |
| Biofilm | ACA32068.1 | HSM_0424 | Myo-inositol catabolism IolB domain protein | 33 | | | | | | 4082925.41 | 1594073.73 |
| Biofilm | ACA32071.1 | HSM_0427 | methylmalonate-semialdehyde dehydrogenase | 54.4 | | | | | | 21854390.67 | 2138552.07 |
| Biofilm | ACA32080.1 | HSM_0435 | Inositol 2-dehydrogenase | 36.4 | | | | | | 26614599.80 | 5514173.44 |
| Biofilm | ACA32085.1 | HSM_0044 | tryptophanyl-tRNA synthetase | 37.5 | | | | | | 13267199.10 | 2523427.66 |
| Biofilm | ACA32094.1 | HSM_0450 | uridine phosphorylase | 27.3 | | | | | | 19371240.77 | 5302536.78 |
| Biofilm | ACA32106.1 | HSM_0461 | Inorganic diphosphatase | 19.3 | | | | | | 2917169.08 | 124015.98 |
| Biofilm | ACA32112.1 | HSM_0467 | leucyl-tRNA synthetase | 98.1 | | | | | | 59260194.41 | 7955078.96 |
| Biofilm | ACA32118.1 | HSM_0472 | aspartate-semialdehyde dehydrogenase | 40.6 | | | | | | 78003923.16 | 21578615.96 |
| Biofilm | ACA32144.1 | HSM_0498 | phosphoribosylformylglycinamidine synthase | 143.1 | | | | | | 72812269.37 | 13103392.70 |
| Biofilm | ACA32151.1 | HSM_0503 | 2C-methyl-D-erythritol 2,4-cyclodiphosphate synthase | 17.3 | | | | | | 3656338.20 | 663193.63 |
| Biofilm | ACA32161.1 | HSM_0512 | Cof-like hydrolase | 30.6 | | | | | | 215289.76 | 89055.45 |
| Biofilm | ACA32165.1 | HSM_0516 | hydrolase, TatD family | 29.4 | | | | | | 5623212.94 | 885038.23 |
| Biofilm | ACA32174.1 | HSM_0524 | lysyl-tRNA synthetase | 56.9 | | | | | | 303061611.58 | 59276908.64 |
| Biofilm | ACA32207.1 | HSM_0556 | Monosaccharide-transporting ATPase | 33.2 | | | | | | 6491914.47 | 3052599.83 |
| Biofilm | ACA32216.1 | HSM_0564 | uroporphyrinogen decarboxylase | 39.3 | | | | | | 1330359.30 | 671011.06 |
| Biofilm | ACA32220.1 | HSM_0568 | glucosamine--fructose-6-phosphate aminotransferase, isomerizing | 67.6 | | | | | | 3406320.58 | 3471577.30 |
| Biofilm | ACA32227.1 | HSM_0576 | Glycerate kinase | 40.1 | | | | | | 796016.40 | 265018.99 |
| Biofilm | ACA32232.1 | HSM_0580 | L-fuculose phosphate aldolase | 24.1 | | | | | | 698993.21 | 111543.22 |
| Biofilm | ACA32235.1 | HSM_0583 | L-fucose isomerase | 65.3 | | | | | | 1222405.80 | 44209.21 |
| Biofilm | ACA32238.1 | HSM_0586 | phosphoglycerate mutase 1 family | 26 | | | | | | 360936936.36 | 94777145.19 |

| | | | | | | | | | | |
|---|---|---|---|---|---|---|---|---|---|---|
| Biofilm | ACA32243.1 | HSM_0590 | putative transcriptional regulator, AsnC family | 17.1 | | | | | 3257435.22 | 1981081.93 |
| Biofilm | ACA32244.1 | HSM_0591 | Aspartate--ammonia ligase | 37.6 | | | | | 1016432.50 | 151530.87 |
| Biofilm | ACA32261.1 | HSM_0608 | amidohydrolase | 42.9 | | | | | 305728450.68 | 55468471.29 |
| Biofilm | ACA32263.1 | HSM_0061 | NusG antitermination factor | 21.1 | | | | | 990516.61 | 788013.74 |
| Biofilm | ACA32264.1 | HSM_0610 | N-6 DNA methylase | 126.6 | | | | | 1209172.76 | 887628.22 |
| Biofilm | ACA32273.1 | HSM_0619 | MraZ protein | 17.2 | | | | | 278154.62 | 38957.25 |
| Biofilm | ACA32291.1 | HSM_0635 | chorismate mutase | 43.9 | | | | | 360911.89 | 417163.39 |
| Biofilm | ACA32360.1 | HSM_0698 | TrkA-N domain protein | 24.5 | | | | | 681794.54 | 482874.50 |
| Biofilm | ACA32363.1 | HSM_0070 | oxaloacetate decarboxylase alpha subunit | 65.5 | | | | | 558304.65 | 477229.60 |
| Biofilm | ACA32373.1 | HSM_0709 | peptide chain release factor 3 | 59.3 | | | | | 1284535.97 | 1132257.65 |
| Biofilm | ACA32377.1 | HSM_0712 | Nucleoside-diphosphate kinase | 16.1 | | | | | 2644621.62 | 2655531.83 |
| Biofilm | ACA32378.1 | HSM_0713 | PepB aminopeptidase | 47.3 | | | | | 12938409.87 | 4847859.70 |
| Biofilm | ACA32381.1 | HSM_0716 | 3-isopropylmalate dehydrogenase | 39.2 | | | | | 1276985.07 | 230877.88 |
| Biofilm | ACA32385.1 | HSM_0072 | transaldolase | 35.1 | | | | | 84302249.76 | 32908937.24 |
| Biofilm | ACA32399.1 | HSM_0732 | succinic semialdehyde dehydrogenase | 53.1 | | | | | 696712.82 | 453879.95 |
| Biofilm | ACA32407.1 | HSM_0074 | fructose-bisphosphate aldolase, class II | 39 | | | | | 227195089.52 | 95599867.18 |
| Biofilm | ACA32411.1 | HSM_0743 | glutamine synthetase, type I | 52.7 | | | | | 104190712.20 | 17941157.26 |
| Biofilm | ACA32413.1 | HSM_0745 | inosine-5'-monophosphate dehydrogenase | 52 | | | | | 10994095.35 | 1125384.71 |
| Biofilm | ACA32432.1 | HSM_0762 | hypoxanthine phosphoribosyltransferase | 20.6 | | | | | 1924457.51 | 917564.64 |
| Biofilm | ACA32433.1 | HSM_0763 | Superoxide dismutase | 23.5 | | | | | 33421365.44 | 9965225.20 |
| Biofilm | ACA32434.1 | HSM_0764 | Malate dehydrogenase (oxaloacetate-decarboxylating) (NADP(+)), Phosphate acetyltransferase | 81.6 | | | | | 151924548.43 | 48254875.69 |
| Biofilm | ACA32435.1 | HSM_0765 | aspartate ammonia-lyase | 51 | | | | | 19311298.84 | 6405736.98 |
| Biofilm | ACA32446.1 | HSM_0775 | alkyl hydroperoxide reductase/ Thiol specific antioxidant/ Mal allergen | 22.3 | | | | | 486216584.20 | 124716569.05 |
| Biofilm | ACA32453.1 | HSM_0781 | succinate dehydrogenase and fumarate reductase iron-sulfur protein | 28.7 | | | | | 1779224.72 | 10105.12 |
| Biofilm | ACA32454.1 | HSM_0782 | fumarate reductase, flavoprotein subunit | 66.2 | | | | | 34074895.17 | 14973978.32 |
| Biofilm | ACA32455.1 | HSM_0783 | lysyl-tRNA synthetase-related protein GenX | 36.7 | | | | | 116639.69 | No Quan Value |
| Biofilm | ACA32461.1 | HSM_0789 | aconitate hydratase 2 | 90.6 | | | | | 8171739.78 | 4849755.19 |

| | | | | | | | | | | |
|---|---|---|---|---|---|---|---|---|---|---|
| Biofilm | ACA32463.1 | HSM_0790 | YCII-related | 11.1 | | | | | 4705119.25 | 2682528.05 |
| Biofilm | ACA32473.1 | HSM_0800 | Haemophilus-specific protein, uncharacterized | 17.2 | | | | | 1397890.46 | 610377.76 |
| Biofilm | ACA32476.1 | HSM_0803 | valyl-tRNA synthetase | 109.5 | | | | | 155702673.50 | 41400030.08 |
| Biofilm | ACA32477.1 | HSM_0804 | tRNA pseudouridine synthase A | 30.6 | | | | | 224140.63 | No Quan Value |
| Biofilm | ACA32490.1 | HSM_0816 | ROK family protein | 32.4 | | | | | 16460748.45 | 5833868.03 |
| Biofilm | ACA32493.1 | HSM_0819 | ketose-bisphosphate aldolase | 32.2 | | | | | 560852.99 | 61276.93 |
| Biofilm | ACA32495.1 | HSM_0820 | protein of unknown function DUF1498 | 26.2 | | | | | 54758.07 | 4571.98 |
| Biofilm | ACA32501.1 | HSM_0826 | mannitol 1-phosphate 5-dehydrogenase | 42.8 | | | | | 4476463.45 | 816793.48 |
| Biofilm | ACA32506.1 | HSM_0832 | Dipeptidase E | 26.2 | | | | | 736676.72 | 147631.59 |
| Biofilm | ACA32512.1 | HSM_0084 | Ribonuclease E inhibitor RraA | 18 | | | | | 1443133.04 | 669890.08 |
| Biofilm | ACA32513.1 | HSM_0840 | phosphoheptose isomerase | 21.6 | | | | | 12245884.64 | 3888063.95 |
| Biofilm | ACA32516.1 | HSM_0843 | RNA polymerase, sigma 70 subunit, RpoD | 71.3 | | | | | 2204386.07 | 1877527.53 |
| Biofilm | ACA32519.1 | HSM_0846 | glycyl-tRNA synthetase, alpha subunit | 34.7 | | | | | 422888.92 | 155402.69 |
| Biofilm | ACA32520.1 | HSM_0847 | conserved hypothetical protein | 10.1 | | | | | 185353.32 | 1458.82 |
| Biofilm | ACA32527.1 | HSM_0853 | conserved hypothetical protein | 38.5 | | | | | 15290434.69 | 6584439.90 |
| Biofilm | ACA32528.1 | HSM_0854 | Methionine synthase vitamin-B12 independent | 38.5 | | | | | 8148110.14 | 2400269.15 |
| Biofilm | ACA32531.1 | HSM_0857 | aspartyl-tRNA synthetase | 66.5 | | | | | 51957807.93 | 23381868.83 |
| Biofilm | ACA32547.1 | HSM_0871 | conserved hypothetical protein | 13.3 | | | | | 2641021.60 | 523438.16 |
| Biofilm | ACA32551.1 | HSM_0875 | hypothetical protein HSM_0875 | 31.4 | | | | | 1932692.90 | 581607.81 |
| Biofilm | ACA32557.1 | HSM_0880 | conserved hypothetical protein | 38.2 | | | | | 178148.23 | No Quan Value |
| Biofilm | ACA32566.1 | HSM_0889 | protein of unknown function UPF0150 | 15.2 | | | | | 1370185.62 | 1139993.11 |
| Biofilm | ACA32608.1 | HSM_0926 | arsenate reductase and related | 13.4 | | | | | 830188.91 | 456603.14 |
| Biofilm | ACA32609.1 | HSM_0927 | succinyl-diaminopimelate desuccinylase | 41.8 | | | | | 4131179.58 | 1486900.62 |
| Biofilm | ACA32611.1 | HSM_0929 | ADP-ribose diphosphatase | 23.3 | | | | | 2203785.27 | 379700.43 |
| Biofilm | ACA32613.1 | HSM_0930 | Calcineurin phosphoesterase domain protein | 32.2 | | | | | 1460353.08 | 788323.26 |
| Biofilm | ACA32619.1 | HSM_0936 | xylose isomerase | 49.6 | | | | | 1201608.29 | 624448.00 |
| Biofilm | ACA32630.1 | HSM_0946 | glutaredoxin, GrxA family | 9.6 | | | | | 10977451.25 | 1427049.20 |
| Biofilm | ACA32641.1 | HSM_0956 | mannose-6-phosphate isomerase, class I | 45.4 | | | | | 14084050.41 | 14993762.37 |

| | | | | | | | | | | |
|---|---|---|---|---|---|---|---|---|---|---|
| Biofilm | ACA32646.1 | HSM_0960 | PTS system, mannose/fructose/sorbose family, IIB subunit | 35.5 | | | | | 291570.37 | 258910.58 |
| Biofilm | ACA32647.1 | HSM_0961 | protein of unknown function DUF496 | 14.6 | | | | | 911982.26 | 838376.44 |
| Biofilm | ACA32648.1 | HSM_0962 | phosphoserine aminotransferase | 40.2 | | | | | 3059125.08 | 1156930.94 |
| Biofilm | ACA32649.1 | HSM_0963 | histidinol-phosphate aminotransferase | 41.6 | | | | | 172958.78 | No Quan Value |
| Biofilm | ACA32657.1 | HSM_0970 | aminotransferase class I and II | 45.4 | | | | | 10398292.85 | 5734602.86 |
| Biofilm | ACA32674.1 | HSM_0988 | Nucleoside-triphosphate--adenylate kinase | 23.7 | | | | | 1489825.84 | 489584.52 |
| Biofilm | ACA32680.1 | HSM_0993 | Endoribonuclease L-PSP | 13.2 | | | | | 10488382.49 | 2682321.90 |
| Biofilm | B0URH4.1 | HSM_0409 | Uronate isomerase | 54 | | | | | 171880904.61 | 32573020.47 |
| Biofilm | B0URI5.1 | HSM_0420 | Triosephosphate isomerase | 26.9 | | | | | 51628324.58 | 24265839.84 |
| Biofilm | B0URI7.1 | HSM_0422 | Phosphopantetheine adenylyltransferase | 17.7 | | | | | 2244251.09 | 746794.74 |
| Biofilm | B0URJ9.1 | HSM_0434 | Inosose dehydratase | 33.6 | | | | | 2015746.42 | 184596.26 |
| Biofilm | B0URN5.1 | HSM_0473 | Phosphoribosylformylglycinamidine cyclo-ligase | 37.1 | | | | | 824251.73 | 203363.69 |
| Biofilm | B0URV5.1 | HSM_0510 | Fe/S biogenesis protein NfuA | 21.4 | | | | | 24284152.00 | No Quan Value |
| Biofilm | B0URX4.1 | HSM_0529 | 5'-methylthioadenosine/S-adenosylhomocysteine nucleosidase | 24 | | | | | 7477138.53 | 1858814.02 |
| Biofilm | B0URX8.1 | HSM_0533 | 50S ribosomal protein L31 | 7.8 | | | | | 619513.77 | 622138.73 |
| Biofilm | B0US06.1 | HSM_0563 | ATP-dependent protease subunit HslV | 19 | | | | | 13495718.52 | 4945766.56 |
| Biofilm | B0US46.1 | HSM_0607 | 50S ribosomal protein L9 | 15.9 | | | | | 5980578.33 | 3595500.91 |
| Biofilm | B0US69.1 | HSM_0630 | D-alanine--D-alanine ligase | 33.9 | | | | | 459062.71 | 22206.39 |
| Biofilm | B0USG9.1 | HSM_0730 | Histidine--tRNA ligase | 48.2 | | | | | 38681392.12 | 17600838.59 |
| Biofilm | B0USH2.1 | HSM_0733 | Gamma-glutamyl phosphate reductase | 45.6 | | | | | 1076858.55 | 865145.45 |
| Biofilm | B0USI5.1 | HSM_0746 | GMP synthase [glutamine-hydrolyzing] | 58.3 | | | | | 834097.84 | 1005348.93 |
| Biofilm | B0USJ0.1 | HSM_0751 | Chaperone protein HtpG | 71.6 | | | | | 202604041.45 | 164972645.59 |
| Biofilm | B0USJ3.1 | HSM_0754 | Glutamate--tRNA ligase | 54.9 | | | | | 7018255.58 | 3984918.96 |
| Biofilm | B0USK5.1 | HSM_0766 | Co-chaperonin GroES | 10.3 | | | | | 139197295.36 | 51767133.62 |
| Biofilm | B0USL2.1 | HSM_0773 | 3-octaprenyl-4-hydroxybenzoate carboxy-lyase | 55.6 | | | | | 1106369.63 | 765319.40 |
| Biofilm | B0UST3.1 | HSM_0850 | Glutamate-1-semialdehyde 2,1-aminomutase | 46.7 | | | | | 3127912.25 | 1359595.25 |

| | | | | | | | | | | |
|---|---|---|---|---|---|---|---|---|---|---|
| Biofilm | B0USU2.1 | HSM_0859 | Bifunctional protein FolD; Includes: Methylenetetrahydrofolate dehydrogenase and Methenyltetrahydrofolate cyclohydrolase | 30.6 | | | | | 503426.52 | 484588.42 |
| Biofilm | B0UT65.1 | HSM_0984 | Uridine kinase | 24.5 | | | | | 347947.92 | 202057.38 |
| Biofilm | B0UT66.1 | HSM_0985 | dCTP deaminase | 21.4 | | | | | 2698211.88 | 311344.92 |
| Biofilm | B0UT70.1 | HSM_0989 | Protein GrpE | 22 | | | | | 27370537.91 | 14042344.26 |
| Biofilm | B0UT99.1 | HSM_1018 | Elongation factor Ts | 30.4 | | | | | 20256157.32 | 5329813.66 |
| Biofilm | B0UTE1.1 | HSM_1060 | Alanine--tRNA ligase | 96.4 | | | | | 2479515.43 | 2237283.55 |
| Biofilm | B0UTI0.1 | HSM_1099 | UPF0234 protein HSM_1099 | 18.6 | | | | | 4590798.37 | 2376938.52 |
| Biofilm | B0UTI6.1 | HSM_1105 | D-aminoacyl-tRNA deacylase | 15.9 | | | | | 61802.42 | 12142.50 |
| Biofilm | B0UTI7.1 | HSM_1106 | N-acetylneuraminate lyase | 32.3 | | | | | 13975120.16 | 4080162.70 |
| Biofilm | B0UTS2.1 | HSM_1197 | Phosphoglucosamine mutase | 47.4 | | | | | 2312280.62 | 1576472.32 |
| Biofilm | B0UTU8.1 | HSM_1223 | Asparagine--tRNA ligase | 52.8 | | | | | 10100950.06 | 3560424.62 |
| Biofilm | B0UU16.1 | HSM_1293 | 5-methyltetrahydropteroyltriglutamate--homocysteine methyltransferase | 85.8 | | | | | 78004860.21 | 21904669.09 |
| Biofilm | B0UU22.1 | HSM_1299 | 6,7-dimethyl-8-ribityllumazine synthase | 16.5 | | | | | 103506121.06 | 17647966.07 |
| Biofilm | B0UU27.1 | HSM_1304 | 4-hydroxy-tetrahydrodipicolinate reductase | 29.2 | | | | | 11075775.02 | 3514343.78 |
| Biofilm | B0UU57.1 | HSM_1334 | Phenylalanine--tRNA ligase alpha subunit | 38 | | | | | 449791.80 | 31363.40 |
| Biofilm | B0UU60.1 | HSM_1337 | Integration host factor subunit alpha | 10.9 | | | | | 5161747.13 | 789199.29 |
| Biofilm | B0UU87.1 | HSM_1366 | Glucose-1-phosphate adenylyltransferase | 49.4 | | | | | 3358161.89 | 1622771.11 |
| Biofilm | B0UUD2.1 | HSM_1411 | Pyridoxal kinase PdxY | 31.3 | | | | | 34362940.98 | 10625033.95 |
| Biofilm | B0UUE2.1 | HSM_1421 | 2-dehydro-3-deoxyphosphooctonate aldolase | 31.2 | | | | | 1330500.32 | 346637.26 |
| Biofilm | B0UUE6.1 | HSM_1425 | Peptide chain release factor 1 | 40.8 | | | | | 342984.75 | No Quan Value |
| Biofilm | B0UUF1.1 | HSM_1430 | Arginine--tRNA ligase | 65.1 | | | | | 61900626.77 | 14350412.74 |
| Biofilm | B0UUF3.1 | HSM_1432 | Succinate--CoA ligase [ADP-forming] subunit beta | 42.5 | | | | | 5314063.18 | 464960.94 |
| Biofilm | B0UUJ3.1 | HSM_1472 | Phosphoenolpyruvate carboxylase | 99.8 | | | | | 580467.01 | 637319.22 |
| Biofilm | B0UUL6.1 | HSM_1495 | Protein-methionine-sulfoxide reductase catalytic subunit MsrP; Flags: Precursor | 36.2 | | | | | 335926.40 | 12945.88 |
| Biofilm | B0UUN2.1 | HSM_1505 | Glucosamine-6-phosphate deaminase | 30.4 | | | | | 15152644.67 | 3831641.40 |

| | | | | | | | | | | |
|---|---|---|---|---|---|---|---|---|---|---|
| Biofilm | B0UUN5.1 | HSM_1508 | Cyclic pyranopterin monophosphate synthase | 17.2 | | | | | 11442070.02 | 2397627.62 |
| Biofilm | B0UUR6.1 | HSM_1539 | Malate dehydrogenase | 32.6 | | | | | 42484633.48 | 16292582.87 |
| Biofilm | B0UUT2.1 | HSM_1555 | RNA chaperone ProQ | 23.4 | | | | | 73978.71 | 12840.51 |
| Biofilm | B0UUT7.1 | HSM_1560 | Serine--tRNA ligase | 48.1 | | | | | 2977932.43 | 3174918.72 |
| Biofilm | B0UUU9.1 | HSM_1572 | tRNA-2-methylthio-N(6)-dimethylallyladenosine synthase | 53.3 | | | | | 473954.55 | 157012.77 |
| Biofilm | B0UUW5.1 | HSM_0007 | Deoxyuridine 5'-triphosphate nucleotidohydrolase | 16.3 | | | | | 12059495.94 | 5853206.69 |
| Biofilm | B0UUZ3.1 | HSM_0035 | Acyl carrier protein | 8.6 | | | | | 13934185.04 | 7753987.98 |
| Biofilm | B0UV12.1 | HSM_0054 | Isoleucine--tRNA ligase | 105.9 | | | | | 147795566.94 | 48943470.86 |
| Biofilm | B0UV33.1 | HSM_0075 | Phosphoglycerate kinase | 41.2 | | | | | 156843159.60 | 54348001.37 |
| Biofilm | B0UV57.1 | HSM_0099 | Sugar fermentation stimulation protein homolog | 27 | | | | | 651192.42 | 217198.08 |
| Biofilm | B0UVH6.1 | HSM_0115 | Cysteine--tRNA ligase | 52.5 | | | | | 5237115.50 | 3474986.81 |
| Biofilm | B0UVH9.1 | HSM_0118 | 50S ribosomal protein L21 | 11.5 | | | | | 1032784.16 | 603008.60 |
| Biofilm | B0UVM2.1 | HSM_0161 | Purine nucleoside phosphorylase DeoD-type | 25.9 | | | | | 18647924.18 | 6791524.61 |
| Biofilm | B0UVY2.1 | HSM_1763 | Probable transcriptional regulatory protein HSM_1763 | 26.2 | | | | | 673046.07 | 263503.31 |
| Biofilm | B0UW05.1 | HSM_1786 | ATP-dependent 6-phosphofructokinase | 35.2 | | | | | 3052559.92 | 1025072.32 |
| Biofilm | B0UW09.2 | HSM_0204 | Bifunctional protein GlmU; Includes: UDP-N-acetylglucosamine pyrophosphorylase and Glucosamine-1-phosphate N-acetyltransferase | 49.4 | | | | | 13554790.81 | 6549920.05 |
| Biofilm | B0UW18.1 | HSM_0213 | Dihydroxy-acid dehydratase | 65.4 | | | | | 76762.24 | No Quan Value |
| Biofilm | B0UW20.1 | HSM_0215 | ATP-dependent Clp protease proteolytic subunit | 21.4 | | | | | 2031592.93 | 479441.75 |
| Biofilm | B0UW56.1 | HSM_0251 | Phosphoribosylaminoimidazole-succinocarboxamide synthase | 32.3 | | | | | 2874361.09 | 366007.12 |
| Biofilm | B0UW60.1 | HSM_0255 | 3-hydroxyacyl-[acyl-carrier-protein] dehydratase FabZ | 17 | | | | | 17328169.81 | 1512259.63 |
| Biofilm | B0UW86.1 | HSM_0281 | Uracil phosphoribosyltransferase | 22.6 | | | | | 1146325.86 | 895773.07 |
| Biofilm | B0UWA8.1 | HSM_1791 | Autonomous glycyl radical cofactor | 14.3 | | | | | 7249305.89 | 3491754.94 |
| Biofilm | B0UWA9.1 | HSM_1792 | Glycine--tRNA ligase beta subunit | 75.4 | | | | | 1458856.19 | 1316904.26 |

| | | | | | | | | | | |
|---|---|---|---|---|---|---|---|---|---|---|
| Biofilm | B0UWG4.1 | HSM_1849 | ATP synthase epsilon chain | 15.7 | | | | | 354324.84 | 195977.38 |
| Biofilm | B0UWH5.1 | HSM_1860 | Cell division protein ZapB | 8.7 | | | | | 713382.07 | 870.27 |
| Biofilm | B0UWL1.1 | HSM_0315 | Protein RecA | 38.1 | | | | | 206481.84 | 108803.08 |
| Biofilm | B0UWM2.1 | HSM_0326 | Ribonuclease PH | 25.8 | | | | | 664547.07 | 306190.15 |
| Biofilm | B0UWS8.1 | HSM_0382 | Serine hydroxymethyltransferase | 45.6 | | | | | 28594502.75 | 9949452.34 |
| Biofilm | B0UWZ5.1 | HSM_1934 | Peptide deformylase | 19.3 | | | | | 5027494.79 | 5232647.25 |
| Biofilm | B0UX14.1 | HSM_1953 | 50S ribosomal protein L4 | 22 | | | | | 647104.50 | 139105.20 |
| Biofilm | B0UX25.1 | HSM_1964 | 50S ribosomal protein L24 | 11.2 | | | | | 8212370.80 | 3397383.95 |
| Biofilm | B0UX29.1 | HSM_1968 | 50S ribosomal protein L6 | 19 | | | | | 1281811.76 | 53157.17 |
| Biofilm | B0UX38.1 | HSM_1977 | 30S ribosomal protein S4 | 23.5 | | | | | 535982.84 | 624458.14 |
| Biofilm | CAV20865.1 | HSM_0032 | unnamed protein product | 34.2 | | | | | 4969783.02 | 1050447.53 |
| Biofilm | CAV21854.1 | HSM_1388 | unnamed protein product | 24.9 | | | | | 5731116.85 | 1681170.60 |
| Biofilm | CAV22576.1 | HSM_0438 | unnamed protein product | 81.8 | | | | | 714819881.57 | 14921861.35 |
| Biofilm | CAV25160.1 | HSM_0855 | unnamed protein product | 17.3 | | | | | 782897.32 | 357055.73 |
| Biofilm | CAY05760.1 | HSM_1850, HSM_1851 | unnamed protein product: ATP synthase F1, beta subunit and gamma subunit | 51.5 | | | | | 41705344.00 | 10535289.29 |
| Biofilm | CAY08832.1 | HSM_1081 | unnamed protein product | 17.4 | | | | | 628555.81 | 282842.21 |
| Biofilm | CAY33861.1 | HSM_1041 | unnamed protein product | 86.7 | | | | | 66261738.00 | 30644426.91 |
| Biofilm | CAY35004.1 | HSM_1307 | unnamed protein product | 44.3 | | | | | 60976128.57 | 18427693.82 |
| Biofilm | CAY36147.1 | HSM_0479 | unnamed protein product | 26 | | | | | 4019027.12 | 2466142.97 |
| Biofilm | CBF76861.1 | HSM_0761 | unnamed protein product | 12 | | | | | 13180282.18 | 4689946.39 |
| Biofilm | CBG07608.1 | HSM_0034 | unnamed protein product | 25.2 | | | | | 125569.48 | 53422.49 |
| Biofilm | CBL85769.1 | HSM_0114 | unnamed protein product | 18.7 | | | | | 23147663.31 | 5142019.70 |
| Biofilm | CBM35200.1 | HSM_1065 | unnamed protein product | 39.7 | | | | | 196909.38 | 177732.22 |
| Biofilm | CBM38073.1 | HSM_0067 | unnamed protein product | 95.4 | | | | | 29556525.02 | 18235327.66 |
| Biofilm | CBV14742.1 | HSM_1256 | unnamed protein product | 37.4 | | | | | 3408790.77 | 1955259.00 |
| Proteins detected under two growth conditions | | | | | | | | | | |
| OMV_Fe & OMV_EDDHA | ACA30758.1 | HSM_1044 | conserved hypothetical protein | 13.1 | 12219526.66 | 331117.58 | 6322717.28 | 2668878.56 | | |
| OMV_Fe & OMV_EDDHA | ACA30793.1 | HSM_1078 | Haemophilus-specific protein, uncharacterized | 17.8 | 20590979.78 | 11014503.29 | 14999603.49 | 8571895.87 | | |

| | | | | | | | | | | |
|---|---|---|---|---|---|---|---|---|---|---|
| OMV_Fe & OMV_EDDHA | ACA30807.1 | HSM_1090 | filamentous haemagglutinin family outer membrane protein | 192.5 | 3594180.80 | 2482101.11 | 918313.37 | 935115.17 | | |
| OMV_Fe & OMV_EDDHA | ACA30938.1 | HSM_1213 | outer membrane efflux protein | 51.8 | 96037824.14 | 42800309.09 | 66507291.66 | 23863768.97 | | |
| OMV_Fe & OMV_EDDHA | ACA31085.1 | HSM_0135 | Tol-Pal system beta propeller repeat TolB | 46.3 | 447823310.59 | 289058261.45 | 240326348.33 | 59355903.98 | | |
| OMV_Fe & OMV_EDDHA | ACA31109.1 | HSM_1371 | peptidase M16 domain protein | 106.5 | 12064294.06 | 1843071.41 | 188998048.31 | 43511957.39 | | |
| OMV_Fe & OMV_EDDHA | ACA31110.1 | HSM_1372 | TonB-dependent receptor | 90.5 | 43106244.60 | 24380213.60 | 764945595.23 | 104362503.16 | | |
| OMV_Fe & OMV_EDDHA | ACA31172.1 | HSM_1428 | conserved hypothetical protein | 11.7 | 27962708.16 | 8453326.64 | 10965581.50 | 2310536.70 | | |
| OMV_Fe & OMV_EDDHA | ACA31225.1 | HSM_1476 | outer membrane lipoprotein LolB | 24.2 | 2448775.03 | 1097043.40 | 2915415.54 | 368214.21 | | |
| OMV_Fe & OMV_EDDHA | ACA31299.1 | HSM_1542 | YadA domain protein | 246.2 | 10265745.60 | 3579030.74 | 2161428.95 | 552407.28 | | |
| OMV_Fe & OMV_EDDHA | ACA31315.1 | HSM_1557 | conserved hypothetical protein | 55.2 | 33392525.89 | 8218649.20 | 16308524.98 | 109674.02 | | |
| OMV_Fe & OMV_EDDHA | ACA31324.1 | HSM_1565 | protein of unknown function DUF1007 | 24.7 | 3522812.12 | 2957174.77 | 800268.84 | 498316.70 | | |
| OMV_Fe & OMV_EDDHA | ACA31331.1 | HSM_1571 | YadA domain protein | 410 | 267225.63 | 34163.29 | No Quan Value | No Quan Value | | |
| OMV_Fe & OMV_EDDHA | ACA31420.1 | HSM_1651 | filamentous haemagglutinin family outer membrane protein | 299.3 | 4084572.77 | 2047339.75 | 5996688.24 | 228475.99 | | |
| OMV_Fe & OMV_EDDHA | ACA31498.1 | HSM_1721 | Haemophilus-specific protein, uncharacterized | 19.6 | 3963732.45 | 3204708.04 | 1515617.59 | 589129.12 | | |
| OMV_Fe & OMV_EDDHA | ACA31563.1 | HSM_1780 | conserved hypothetical protein | 22.9 | 6708195.34 | 1756078.65 | 2848224.50 | 101850.18 | | |
| OMV_Fe & OMV_EDDHA | ACA31571.1 | HSM_1788 | conserved hypothetical protein | 21.8 | 38685633.01 | 43419688.53 | 12835855.25 | 7195922.26 | | |
| OMV_Fe & OMV_EDDHA | ACA31602.1 | HSM_1815 | conserved hypothetical protein | 16.1 | 117797974.52 | 109596172.60 | 59730503.31 | 40359057.40 | | |
| OMV_Fe & OMV_EDDHA | ACA31628.1 | HSM_1840 | ApbE family lipoprotein | 38.5 | 517962.16 | 222415.75 | 347180.16 | No Quan Value | | |
| OMV_Fe & OMV_EDDHA | ACA31757.1 | HSM_1962 | TonB-dependent receptor | 98.3 | 131157186.45 | 56430400.86 | 62031325.86 | 1819424.06 | | |
| OMV_Fe & OMV_EDDHA | ACA31785.1 | HSM_1988 | TonB-dependent lactoferrin and transferrin receptor | 84.6 | 266098477.22 | 105727873.45 | 1180589809.19 | 109555159.93 | | |

| | | | | | | | | | | |
|---|---|---|---|---|---|---|---|---|---|---|
| OMV_Fe & OMV_EDDHA | ACA31786.1 | HSM_1989 | conserved hypothetical protein | 17.8 | 51576061.98 | 30901353.18 | 30137485.21 | 9949836.63 | | |
| OMV_Fe & OMV_EDDHA | ACA31806.1 | HSM_2005 | conserved hypothetical protein | 22.3 | 10800463.72 | 3731597.28 | 5029616.76 | 2448659.34 | | |
| OMV_Fe & OMV_EDDHA | ACA31833.1 | HSM_0210 | surface antigen (D15) | 67.5 | 14186805.48 | 8108058.86 | 4747360.51 | 364499.29 | | |
| OMV_Fe & OMV_EDDHA | ACA31902.1 | HSM_0273 | hypothetical protein HSM_0273 | 15.2 | 33050767.16 | 21546081.54 | 17243773.21 | 10798932.04 | | |
| OMV_Fe & OMV_EDDHA | ACA31904.1 | HSM_0275 | hypothetical protein HSM_0275 | 13.4 | 3133827.85 | 2967074.60 | 986398.56 | 139072.49 | | |
| OMV_Fe & OMV_EDDHA | ACA31905.1 | HSM_0276 | hypothetical protein HSM_0276 | 13.1 | 3774677.70 | 3330276.90 | 3225353.23 | 1655667.44 | | |
| OMV_Fe & OMV_EDDHA | ACA31995.1 | HSM_0359 | peptidase U6 penicillin-insensitive murein endopeptidase | 31.5 | 7318971.58 | 5743457.09 | 1922315.39 | 468279.96 | | |
| OMV_Fe & OMV_EDDHA | ACA32002.1 | HSM_0365 | type IV pilus biogenesis/stability protein PilW | 20.7 | 4835284.53 | 1693777.97 | 1878561.13 | 201563.61 | | |
| OMV_Fe & OMV_EDDHA | ACA32043.1 | HSM_0401 | conserved hypothetical protein | 24 | 2199470.80 | 1430756.27 | 1441454.95 | 1010.75 | | |
| OMV_Fe & OMV_EDDHA | ACA32046.1 | HSM_0404 | sigma E regulatory protein, MucB/RseB | 36.3 | 1960262.16 | 1498254.52 | 1788551.27 | 604836.10 | | |
| OMV_Fe & OMV_EDDHA | ACA32059.1 | HSM_0416 | conserved hypothetical protein | 19.8 | 97388422.11 | 75240269.04 | 42616184.41 | 20065323.54 | | |
| OMV_Fe & OMV_EDDHA | ACA32105.1 | HSM_0460 | peptidase M48 Ste24p | 27.9 | 6268327.17 | 6149430.60 | 5626219.54 | 3114608.61 | | |
| OMV_Fe & OMV_EDDHA | ACA32115.1 | HSM_0047 | TonB-dependent receptor | 102.6 | 47037477.29 | 24327799.50 | 2469711909.02 | 238717428.42 | | |
| OMV_Fe & OMV_EDDHA | ACA32122.1 | HSM_0476 | membrane protein involved in aromatic hydrocarbon degradation | 43.4 | 7064332.73 | 2449946.17 | 743145.38 | 489361.66 | | |
| OMV_Fe & OMV_EDDHA | ACA32170.1 | HSM_0520 | conserved hypothetical protein | 21.9 | 70577480.32 | 52050282.14 | 32239597.29 | 14190628.64 | | |
| OMV_Fe & OMV_EDDHA | ACA32176.1 | HSM_0526 | thiol:disulfide interchange protein | 26.2 | 79982665.71 | 23175961.34 | 40592662.87 | 6826327.33 | | |
| OMV_Fe & OMV_EDDHA | ACA32230.1 | HSM_0579 | conserved hypothetical protein | 10.8 | 38221946.71 | 21333367.54 | 11990700.66 | 9340695.84 | | |
| OMV_Fe & OMV_EDDHA | ACA32241.1 | HSM_0589 | protein of unknown function DUF610 YibQ | 32 | 2823674.86 | 2566282.36 | 501286.59 | 397203.71 | | |
| OMV_Fe & OMV_EDDHA | ACA32277.1 | HSM_0622 | Peptidoglycan glycosyltransferase | 67.3 | 8109806.11 | 3136822.96 | 6015804.19 | 2086365.28 | | |

| | | | | | | | | | | |
|---|---|---|---|---|---|---|---|---|---|---|
| OMV_Fe & OMV_EDDHA | ACA32300.1 | HSM_0643 | conserved hypothetical protein | 19.5 | 262160425.25 | 249124974.40 | 94677641.33 | 32816159.64 | | |
| OMV_Fe & OMV_EDDHA | ACA32369.1 | HSM_0705 | TRAP transporter solute receptor, TAXI family | 36 | 396018.81 | 81544.26 | 1761487.09 | 1706045.69 | | |
| OMV_Fe & OMV_EDDHA | ACA32372.1 | HSM_0708 | YadA domain protein | 388.2 | 4551462.20 | 29039.16 | 917361.65 | 690259.95 | | |
| OMV_Fe & OMV_EDDHA | ACA32417.1 | HSM_0749 | transferrin binding protein | 71.3 | 31031393.02 | 17703814.24 | 75262942.83 | 21195083.35 | | |
| OMV_Fe & OMV_EDDHA | ACA32421.1 | HSM_0752 | conserved hypothetical protein | 9.6 | 31258463.47 | 25123426.42 | 17843621.01 | 1682690.99 | | |
| OMV_Fe & OMV_EDDHA | ACA32440.1 | HSM_0077 | YadA domain protein | 425.6 | 529987.62 | 155147.94 | 108509.65 | 122431.18 | | |
| OMV_Fe & OMV_EDDHA | ACA32468.1 | HSM_0797 | Haemophilus-specific protein, uncharacterized | 17 | 170665.92 | No Quan Value | 231665.20 | No Quan Value | | |
| OMV_Fe & OMV_EDDHA | ACA32489.1 | HSM_0815 | MltA domain protein | 39.5 | 57174674.92 | 32299248.43 | 41549374.69 | 106229.91 | | |
| OMV_Fe & OMV_EDDHA | ACA32621.1 | HSM_0938 | Haemagluttinin domain protein | 39.2 | 49239594.58 | 3664078.18 | 55520311.20 | 3441998.42 | | |
| OMV_Fe & OMV_EDDHA | ACA32627.1 | HSM_0943 | 4-phytase | 64.4 | 2033369.49 | 1153560.61 | 277139.53 | 32345.70 | | |
| OMV_Fe & OMV_EDDHA | ACA32638.1 | HSM_0953 | Haemagluttinin domain protein | 41.3 | 72621371.39 | 19498688.06 | 25110211.79 | 5122168.54 | | |
| OMV_Fe & OMV_EDDHA | B0USI0.1 | HSM_0741 | 30S ribosomal protein S20 | 9.6 | 3502566.70 | 1363889.17 | 4733133.89 | 3190287.02 | | |
| OMV_Fe & OMV_EDDHA | B0UUL1.1 | HSM_1490 | Outer membrane transporter protein IbpB; Flags: Precursor | 66.3 | 113532789.18 | 54243929.52 | 56596702.58 | 9434504.03 | | |
| OMV_Fe & OMV_EDDHA | B0UWE6.1 | HSM_1831 | Membrane-bound lytic murein transglycosylase C; Flags: Precursor | 40.8 | 113144413.58 | 81244612.33 | 53065020.01 | 16508319.49 | | |
| OMV_Fe & OMV_EDDHA | B0UX18.1 | HSM_1957 | 50S ribosomal protein L22 | 12.2 | 10288916.62 | 1433513.96 | 3287873.14 | 215327.18 | | |
| OMV_Fe & OMV_EDDHA | B0UX30.1 | HSM_1969 | 50S ribosomal protein L18 | 12.9 | 1496078.84 | 1243066.85 | 61773.70 | 36482.12 | | |
| OMV_Fe & OMV_EDDHA | CBN71109.1 | HSM_1266 | unnamed protein product | 49 | 86927246.99 | 33915629.95 | 17127977.43 | 439259.78 | | |
| OMV_Fe & Biofilm | ACA30946.1 | HSM_1220 | ribosomal protein S9 | 14.7 | 1797144.58 | 1174015.77 | | | 3078886.53 | 1508615.10 |
| OMV_Fe & Biofilm | ACA31378.1 | HSM_1613 | Phosphopyruvate hydratase | 45.8 | 222677.19 | No Quan Value | | | 486519635.86 | 67264436.18 |

| | | | | | | | | | | |
|---|---|---|---|---|---|---|---|---|---|---|
| OMV_Fe & Biofilm | ACA31705.1 | HSM_1915 | TRAP dicarboxylate transporter, DctP subunit | 37.5 | 1923452.41 | No Quan Value | | | 5863483.48 | 1035080.74 |
| OMV_Fe & Biofilm | ACA31760.1 | HSM_1965 | ribosomal protein L5 | 20.3 | 3754036.75 | 5158625.75 | | | 847272.98 | 155552.18 |
| OMV_Fe & Biofilm | ACA31771.1 | HSM_1975 | ribosomal protein S13 | 13.2 | 1054524.01 | 227250.86 | | | 3031849.78 | 1577111.15 |
| OMV_Fe & Biofilm | ACA32190.1 | HSM_0540 | glyceraldehyde-3-phosphate dehydrogenase, type I | 35.5 | No Quan Value | No Quan Value | | | 691682272.53 | 302620618.64 |
| OMV_Fe & Biofilm | ACA32204.1 | HSM_0553 | DNA-directed RNA polymerase, beta subunit | 149.9 | 285824.27 | 178340.62 | | | 150468422.02 | 64361306.35 |
| OMV_Fe & Biofilm | ACA32205.1 | HSM_0554 | DNA-directed RNA polymerase, beta' subunit | 157.8 | 2231187.98 | 1165489.45 | | | 109350849.55 | 38989529.21 |
| OMV_Fe & Biofilm | ACA32218.1 | HSM_0566 | histone family protein DNA-binding protein | 9.3 | 1172841.14 | 1382645.29 | | | 40813978.38 | 7458413.86 |
| OMV_Fe & Biofilm | B0URQ3.1 | HSM_0493 | Periplasmic nitrate reductase; Flags: Precursor | 93 | 372723.15 | 163999.71 | | | 40530533.77 | 12498027.87 |
| OMV_Fe & Biofilm | B0UTU4.1 | HSM_1219 | 50S ribosomal protein L13 | 15.9 | 2591436.01 | 2686793.18 | | | 823878.03 | 262058.86 |
| OMV_Fe & Biofilm | B0UWC5.1 | HSM_1808 | 30S ribosomal protein S7 | 17.7 | 510746.19 | 140093.19 | | | 7949092.09 | 3384412.24 |
| OMV_Fe & Biofilm | B0UX20.1 | HSM_1959 | 50S ribosomal protein L16 | 15.2 | 844794.52 | 777438.65 | | | 180321.86 | 158612.34 |
| OMV_Fe & Biofilm | B0UX28.1 | HSM_1967 | 30S ribosomal protein S8 | 14 | 1758827.85 | 1950520.39 | | | 2781700.93 | 1802994.16 |
| OMV_EDDHA & Biofilm | ACA30834.1 | HSM_1114 | Polyribonucleotide nucleotidyltransferase | 77.5 | | | 9765112.98 | No Quan Value | 241223772.51 | 69970272.99 |
| OMV_EDDHA & Biofilm | ACA31090.1 | HSM_1354 | ribosomal protein L20 | 13.4 | | | 1640417.71 | 1400891.64 | 1382328.36 | 944810.22 |
| OMV_EDDHA & Biofilm | ACA31620.1 | HSM_1833 | ketol-acid reductoisomerase | 54.6 | | | 1003307.29 | 1228065.81 | 271274241.25 | 24720761.19 |
| OMV_EDDHA & Biofilm | B0UUD4.1 | HSM_1413 | Glucose-6-phosphate isomerase | 61.9 | | | 1297139.03 | No Quan Value | 435753052.66 | 79994982.95 |
| OMV_EDDHA & Biofilm | CAY37546.1 | HSM_1664 | unnamed protein product | 73.4 | | | 270413.50 | No Quan Value | 408276398.79 | 101301346.66 |
| Common proteins for all three growth conditions | | | | | | | | | | |
| Common | ACA30723.1 | HSM_1012 | 2-oxo-acid dehydrogenase E1 subunit, homodimeric type | 99.7 | 1273879.28 | 247293.59 | 18446685.93 | 22772224.71 | 277986174.46 | 26691256.76 |
| Common | ACA30724.1 | HSM_1013 | pyruvate dehydrogenase complex dihydrolipoamide acetyltransferase | 66.5 | 648558.41 | No Quan Value | 5512689.88 | 7106530.55 | 28063797.78 | 14355945.17 |

| | | | | | | | | | | |
|---|---|---|---|---|---|---|---|---|---|---|
| Common | ACA30725.1 | HSM_1014 | dihydrolipoamide dehydrogenase | 50.8 | 866404.38 | 781336.30 | 4806435.53 | 6515769.46 | 141138520.18 | 26383337.01 |
| Common | ACA30732.1 | HSM_1020 | PpiC-type peptidyl-prolyl cis-trans isomerase | 69.9 | 10031918.37 | 7269381.47 | 17414434.08 | 6962504.20 | No Quan Value | No Quan Value |
| Common | ACA30764.1 | HSM_0105 | periplasmic binding protein/LacI transcriptional regulator | 35.5 | 78726789.40 | 34838232.54 | 47204948.15 | 3701798.50 | 135904923.85 | 57923095.13 |
| Common | ACA30832.1 | HSM_1112 | TRAP dicarboxylate transporter, DctP subunit | 36.5 | 21620127.46 | 15071775.87 | 7950720.00 | 2135251.65 | 8317747.88 | 3472483.27 |
| Common | ACA30928.1 | HSM_1204 | transport-associated | 20.8 | 498314233.78 | 167356466.66 | 293768949.61 | 95254981.40 | 6988019.23 | 2960998.08 |
| Common | ACA30955.1 | HSM_1229 | TRAP dicarboxylate transporter, DctP subunit | 36.2 | 8228642.89 | 3845227.86 | 5177415.66 | 496161.34 | 1646506.81 | 1389649.42 |
| Common | ACA30959.1 | HSM_1232 | ABC-type sugar-binding periplasmic protein | 40 | 11388228.63 | 5318511.73 | 9885577.62 | 96353.12 | 2076670.85 | 245722.81 |
| Common | ACA30961.1 | HSM_1234 | Monosaccharide-transporting ATPase | 33.6 | 9858222.77 | 4733223.97 | 7500918.97 | 506693.47 | 56475326.58 | 23145649.44 |
| Common | ACA30978.1 | HSM_0125 | extracellular solute-binding protein family 3 | 26.4 | 187240271.64 | 65864109.97 | 77437005.74 | 17319144.21 | 81330693.34 | 28778104.51 |
| Common | ACA30979.1 | HSM_1250 | extracellular solute-binding protein family 1 | 37.6 | 1246978148.90 | 579315924.85 | 1308827223.09 | 210055719.10 | 367714723.67 | 187976209.70 |
| Common | ACA31004.1 | HSM_1275 | molybdopterin guanine dinucleotide-containing S/N-oxide reductase | 90.9 | 11165666.93 | 1286701.14 | 7769052.80 | 1333543.65 | 2605631819.04 | 497618456.62 |
| Common | ACA31013.1 | HSM_1283 | outer membrane lipoprotein carrier protein LolA | 23.4 | 451040.43 | 22014.17 | 242542.60 | 14274.76 | No Quan Value | No Quan Value |
| Common | ACA31015.1 | HSM_1285 | protease Do | 49.5 | 525177555.86 | 132943145.48 | 435122492.51 | 154261401.02 | 46612724.36 | 432269.74 |
| Common | ACA31051.1 | HSM_1317 | SmpA/OmlA domain protein | 31 | 1212610086.56 | 710405903.60 | 674475502.60 | 3024719.26 | 5069636.49 | 632150.61 |
| Common | ACA31071.1 | HSM_1335 | conserved hypothetical protein | 18.3 | 50171242.27 | 22055421.78 | 24107043.42 | 8183117.31 | 18221544.85 | 7074726.03 |
| Common | ACA31096.1 | HSM_0136 | Peptidoglycan-associated lipoprotein | 16.5 | 786872350.40 | 35386477.39 | 394899469.30 | 219946874.24 | 4239564.60 | 1270623.42 |
| Common | ACA31171.1 | HSM_1427 | 17 kDa surface antigen | 15.2 | 1877111407.34 | 734759592.13 | 866701289.41 | 346316259.98 | 9506465.66 | 2173350.03 |
| Common | ACA31193.1 | HSM_1447 | porin Gram-negative type | 41.7 | 34171123380.65 | 13229610288.39 | 22006152859.51 | 57381272.10 | 436326345.64 | 11069138.79 |
| Common | ACA31204.1 | HSM_1457 | outer membrane chaperone Skp (OmpH) | 21.5 | 367480178.50 | 68000826.99 | 223429735.96 | 81838092.05 | 135402398.49 | 82342686.33 |
| Common | ACA31205.1 | HSM_1458 | surface antigen (D15) | 89.9 | 4051163261.96 | 844882795.25 | 1843248176.74 | 62194277.63 | 47608358.35 | 10440265.88 |
| Common | ACA31238.1 | HSM_1488 | thiamine ABC transporter, periplasmic binding protein | 38.4 | 15381059.84 | 5191751.76 | 13773125.52 | 3338822.59 | 11819991.74 | 5643251.50 |
| Common | ACA31239.1 | HSM_1489 | cysteine protease domain, YopT-type | 449.8 | 2120520446.86 | 1923564132.12 | 197008124.10 | 55222770.49 | 2136194.36 | 1805066.49 |

| | | | | | | | | | | |
|---|---|---|---|---|---|---|---|---|---|---|
| Common | ACA31267.1 | HSM_1513 | OmpA domain protein transmembrane region-containing protein | 36.7 | 11842198523.30 | 5373843910.42 | 6156465166.71 | 667296061.26 | 119379692.85 | 8300235.55 |
| Common | ACA31288.1 | HSM_1532 | extracellular solute-binding protein family 1 | 39.1 | 12413705.41 | 4772436.33 | 3677653.95 | 1887477.06 | 2917883.65 | 652698.71 |
| Common | ACA31314.1 | HSM_1556 | carboxyl-terminal protease | 76.8 | 210143066.01 | 5462439.94 | 66823374.87 | 660730.74 | 9124323.64 | 7354304.99 |
| Common | ACA31337.1 | HSM_1577 | extracellular solute-binding protein family 1 | 39.9 | 57513889.70 | 9204172.52 | 30116090.08 | 6020851.57 | 5765658.27 | 2345982.36 |
| Common | ACA31348.1 | HSM_1587 | toluene tolerance family protein | 24 | 89015159.01 | 44036352.49 | 41186228.54 | 7735087.84 | 27228181.61 | 10112106.11 |
| Common | ACA31418.1 | HSM_0165 | ribosomal protein S16 | 9.2 | 13814259.45 | 7436137.21 | 6149158.56 | 3725462.03 | 1413918.88 | 557410.38 |
| Common | ACA31465.1 | HSM_1692 | conserved hypothetical protein | 15.7 | 16978718.98 | 7222038.87 | 7852617.27 | 6310695.41 | 56589378.31 | 26893404.72 |
| Common | ACA31502.1 | HSM_1725 | protein of unknown function DUF411 | 16.7 | 71156592.32 | 39579475.20 | 3761510.63 | 4168787.14 | 59412871.46 | 21838151.61 |
| Common | ACA31503.1 | HSM_1726 | multicopper oxidase type 3 | 58.6 | 172231512.56 | 8850210.20 | 56520257.39 | 17431514.03 | 318221100.19 | 54466975.02 |
| Common | ACA31508.1 | HSM_1730 | multicopper oxidase type 3 | 58.7 | 2602548.91 | 1210859.72 | 217905.28 | No Quan Value | 14277561.40 | 5385206.84 |
| Common | ACA31585.1 | HSM_0180 | DSBA oxidoreductase | 23 | 41768346.29 | 35867547.75 | 11188777.04 | 3899697.93 | 8523473.68 | 3810275.55 |
| Common | ACA31593.1 | HSM_1807 | translation elongation factor G | 77.1 | 614082.64 | 515490.32 | 1407031.16 | 1563348.75 | 544652560.55 | 166317117.87 |
| Common | ACA31604.1 | HSM_1817 | VacJ family lipoprotein | 28.3 | 130386467.89 | 62625856.91 | 66352096.39 | 14040893.92 | 76600.67 | No Quan Value |
| Common | ACA31613.1 | HSM_1827 | protein of unknown function DUF302 | 16.1 | 238115838.08 | 85610028.34 | 58880105.70 | 16676833.49 | 90808839.12 | 38845159.66 |
| Common | ACA31622.1 | HSM_1835 | trimethylamine-N-oxide reductase TorA | 93.6 | 115756.84 | 12479.09 | 9151349.28 | 4300824.23 | 155477376.98 | 32487297.69 |
| Common | ACA31627.1 | HSM_0184 | Sel1 domain protein repeat-containing protein | 27.6 | 46677540.50 | 16282753.99 | 25311243.56 | 3351731.33 | 4070802.28 | 1917023.15 |
| Common | ACA31633.1 | HSM_1845 | NADH:ubiquinone oxidoreductase, subunit C | 27.6 | 852961.59 | 274606.47 | 1169932.06 | No Quan Value | 194147.53 | 122623.70 |
| Common | ACA31714.1 | HSM_1923 | sugar ABC transporter, sugar-binding protein | 35 | 19001704.27 | 11153018.92 | 6143502.56 | 1486659.13 | 21660040.79 | 7420098.01 |
| Common | ACA31740.1 | HSM_1947 | ABC transporter, solute-binding, sugar transport | 36.7 | 6020405.78 | 3011070.51 | 3422722.31 | 109632.46 | 16072343.87 | 7068482.55 |
| Common | ACA31758.1 | HSM_1963 | ribosomal protein L14 | 13.5 | 8034818.34 | 7236570.20 | 15264693.66 | 21471624.92 | 8750752.02 | 5414927.16 |
| Common | ACA31787.1 | HSM_0199 | periplasmic solute binding protein | 32.6 | 338617214.31 | 175228181.12 | 2473298807.39 | 210077235.50 | 168821142.84 | 61404409.36 |
| Common | ACA31804.1 | HSM_2003 | conserved hypothetical protein | 15.4 | 715482087.06 | 288396899.73 | 393102671.45 | 157181558.92 | 4905548.93 | 1799153.55 |
| Common | ACA31830.1 | HSM_0208 | conserved hypothetical protein | 26.4 | 82102312.79 | 44128348.08 | 44269441.62 | 1008357.22 | 898646.24 | 942657.63 |
| Common | ACA31914.1 | HSM_0284 | conserved hypothetical protein | 21.7 | 16766386.07 | 6277893.66 | 11877901.56 | 4142774.34 | 1691528.18 | 542704.13 |
| Common | ACA31915.1 | HSM_0285 | Beta-glucuronidase | 68.4 | 1611492.80 | 1304932.73 | 11965412.91 | 15028989.62 | 991742865.63 | 132165535.43 |
| Common | ACA31970.1 | HSM_0336 | conserved hypothetical protein | 28.4 | 646464753.63 | 326061556.93 | 391156632.08 | 11370239.94 | 9646629.57 | 3306956.77 |

| | | | | | | | | | | |
|---|---|---|---|---|---|---|---|---|---|---|
| Common | ACA31971.1 | HSM_0337 | conserved hypothetical protein | 28.4 | 787782027.68 | 427396977.14 | 442602926.70 | 9138646.05 | 10447485.14 | 4562532.14 |
| Common | ACA31973.1 | HSM_0339 | conserved hypothetical protein | 30.4 | 1765528.02 | 1059751.15 | 1833397.91 | 507279.62 | 489273.25 | 72140.98 |
| Common | ACA31977.1 | HSM_0342 | molybdenum ABC transporter, periplasmic molybdate-binding protein | 26.5 | 3231354.44 | 263863.04 | 1785421.05 | No Quan Value | 3240476.90 | 534247.91 |
| Common | ACA31980.1 | HSM_0345 | conserved hypothetical protein | 24.4 | 49839464.43 | 24310095.96 | 33091980.52 | 11144792.78 | 716300.85 | 449339.43 |
| Common | ACA32007.1 | HSM_0037 | ribosomal protein L1 | 24.1 | 4312806.94 | 3791389.40 | 2028467.80 | 621265.63 | 4452239.16 | 375126.99 |
| Common | ACA32073.1 | HSM_0429 | Monosaccharide-transporting ATPase | 33.9 | 12654156.50 | 11808096.31 | 444492.86 | 128121.51 | 1916332.96 | 1296416.88 |
| Common | ACA32109.1 | HSM_0464 | PpiC-type peptidyl-prolyl cis-trans isomerase | 36.1 | 432945749.20 | 123266911.53 | 281235871.28 | 16490828.88 | 1053454.96 | 206326.66 |
| Common | ACA32113.1 | HSM_0468 | Rare lipoprotein B | 18.8 | 189541198.88 | 93902261.10 | 75014765.99 | 11115623.89 | 359041.90 | 240570.76 |
| Common | ACA32145.1 | HSM_0499 | peptidase M23B | 37.9 | 456869823.31 | 222809197.09 | 272634645.69 | 12360625.48 | 9865412.56 | 516945.39 |
| Common | ACA32200.1 | HSM_0055 | conserved hypothetical protein | 20.9 | 54749593.60 | 30190226.53 | 12905981.45 | 8069030.87 | 1516176.48 | 1195227.98 |
| Common | ACA32221.1 | HSM_0569 | lipoprotein, YaeC family | 30.3 | 14902097.24 | 4039407.08 | 8940487.11 | 2689311.00 | 4756017.18 | 1792768.28 |
| Common | ACA32259.1 | HSM_0606 | ribosomal protein S18 | 9 | 7186943.56 | 1137406.36 | 6716660.21 | 961763.84 | 1195248.89 | 532312.19 |
| Common | ACA32292.1 | HSM_0636 | Tetratricopeptide TPR_2 repeat protein | 29.3 | 550979562.89 | 271458867.33 | 430636606.66 | 51090718.97 | 5487391.67 | 1889914.92 |
| Common | ACA32358.1 | HSM_0696 | extracellular solute-binding protein family 5 | 61.2 | 335731028.68 | 573598.04 | 240294195.04 | 56683594.09 | 520875198.88 | 68701498.86 |
| Common | ACA32359.1 | HSM_0697 | extracellular solute-binding protein family 5 | 62.5 | 148302392.46 | 33660295.25 | 107368371.47 | 22974610.13 | 149556174.68 | 28671248.50 |
| Common | ACA32396.1 | HSM_0073 | Organic solvent tolerance protein | 91.1 | 233802325.90 | 59136987.07 | 117535972.80 | 8130251.96 | 255188.40 | 12887.58 |
| Common | ACA32419.1 | HSM_0750 | TonB-dependent lactoferrin and transferrin receptor | 109.6 | 531580027.20 | 198794605.63 | 589033096.64 | 29993476.65 | 124208.35 | 31916.70 |
| Common | ACA32447.1 | HSM_0776 | ribosomal protein S15 | 10.2 | 7311222.20 | 2661718.47 | 1113330.96 | 763990.84 | 1970702.67 | 1540627.65 |
| Common | ACA32460.1 | HSM_0788 | extracellular solute-binding protein family 5 | 58.4 | 41574449.96 | 8791959.17 | 42326729.15 | 12032763.20 | 142583572.72 | 26927164.73 |
| Common | ACA32483.1 | HSM_0081 | extracellular solute-binding protein family 3 | 28.5 | 853604980.58 | 545549524.70 | 67303174.89 | 24354071.16 | 32832159.79 | 12305754.60 |
| Common | ACA32487.1 | HSM_0813 | periplasmic solute binding protein | 44.2 | 265519323.78 | 4181013.82 | 108235540.36 | 19634557.73 | 136630193.35 | 40168649.08 |
| Common | ACA32496.1 | HSM_0821 | periplasmic binding protein/LacI transcriptional regulator | 32.3 | 25519681.02 | 12520368.69 | 15842321.67 | 1803331.27 | 90981265.97 | 42670165.81 |
| Common | ACA32521.1 | HSM_0848 | hypothetical protein HSM_0848 | 14.4 | 242023907.25 | 98327955.89 | 231362572.83 | 33627342.76 | 1108774.73 | 265582.01 |
| Common | ACA32587.1 | HSM_0907 | conserved hypothetical protein | 36.3 | 62177326.80 | 25530783.52 | 212321085.08 | 177856251.62 | 764614.13 | 18696.37 |
| Common | ACA32601.1 | HSM_0092 | Monosaccharide-transporting ATPase | 30.4 | 77207634.67 | 18801760.03 | 32550836.23 | 1897864.38 | 68638546.76 | 31311958.93 |
| Common | ACA32634.1 | HSM_0095 | SmpA/OmlA domain protein | 15.9 | 445048132.97 | 105194011.27 | 354743412.40 | 173871656.76 | 3727810.29 | 883449.89 |

| | | | | | | | | | | |
|---|---|---|---|---|---|---|---|---|---|---|
| Common | ACA32668.1 | HSM_0982 | extracellular solute-binding protein family 1 | 37.7 | 6757797.49 | 1511876.08 | 3970779.43 | 588994.86 | 7803656.97 | 4424575.74 |
| Common | ACA32669.1 | HSM_0983 | extracellular solute-binding protein family 1 | 38.4 | 10408370.48 | 2379070.65 | 6920743.68 | 597144.15 | 16842228.38 | 6651052.16 |
| Common | B0USK6.1 | HSM_0767 | Chaperonin GroEL | 57.4 | 10817953.81 | 2479062.23 | 47723168.62 | 52739939.70 | 1054557304.19 | 75039095.88 |
| Common | B0USS4.1 | HSM_0841 | 30S ribosomal protein S21 | 8.5 | 5888806.23 | 1402379.75 | 983428.00 | 868516.16 | 472942.73 | 161246.25 |
| Common | B0UTT2.1 | HSM_1207 | Penicillin-binding protein activator LpoA; Flags: Precursor | 66.3 | 2000857880.85 | 1086005900.80 | 1075179057.11 | 60440060.40 | 10488903.23 | 5622953.62 |
| Common | B0UUZ6.1 | HSM_0038 | 50S ribosomal protein L10 | 17.6 | 3325136.26 | 1297140.98 | 352988.27 | 323085.08 | 8390506.15 | 41191.49 |
| Common | B0UV21.1 | HSM_0063; HSM_1806 | Elongation factor Tu | 43.4 | 6198604.78 | 286968.15 | 1270783.00 | 694519.28 | 426588380.11 | 259341856.85 |
| Common | B0UVZ0.1 | HSM_1771 | Cell division protein FtsP; Flags: Precursor | 52.3 | 300814.66 | 262435.03 | 699454.38 | 94194.14 | 3749687.06 | 1499863.26 |
| Common | B0UW71.1 | HSM_0266 | UPF0319 protein HSM_0266; Flags: Precursor | 23.6 | 246759731.86 | 93724457.15 | 132902916.86 | 2074276.00 | 14653070.05 | 6355135.01 |
| Common | B0UWR4.1 | HSM_0368 | Chaperone protein DnaK | 68.4 | 987980.33 | 973829.93 | 1303722.97 | 1144125.69 | 3001198259.35 | 474176593.45 |
| Common | B0UX31.1 | HSM_1970 | 30S ribosomal protein S5 | 17.4 | 3815832.38 | 2451095.94 | 1794072.99 | 1974934.57 | 4046454.68 | 3506711.83 |
| Common | B0UX40.1 | HSM_1979 | 50S ribosomal protein L17 | 14.4 | 7974337.90 | 1276357.94 | 7811375.31 | 1014734.16 | 3012898.30 | 1694039.42 |
| Common | CAY37763.1 | HSM_1799 | unnamed protein product | 25.4 | 39701096.26 | 16718890.61 | 21644428.63 | 1127478.62 | 5313523.21 | 1385099.47 |
| Common | CBN71009.1 | HSM_0341 | unnamed protein product | 51.1 | 1624743323.64 | 325505103.18 | 584271797.60 | 66737508.57 | 1742153.56 | 599632.00 |
| Common | CBN71110.1 | HSM_0053 | unnamed protein product | 37.2 | 705916333.93 | 311346848.66 | 457775720.64 | 105546428.01 | 4230760.07 | 1230362.30 |
| Common | DAA01283.1 | HSM_1761 | TPA_exp: putative glycerophosphoryl diester phosphodiesterase | 41.4 | 73489192.39 | 25833363.40 | 41926976.11 | 684232.77 | 620261.38 | 65513.55 |

[a] A protein was counted as present when it was found in both biological replicates of one growth condition and as absent when it was not found in any of the replicates. OMV_Fe: unique proteins in OMVs collected under $Fe(NO_3)_3$ treatment (an iron-sufficient condition); OMV_EDDHA: unique proteins in OMVs collected under EDDHA treatment (an iron-deficient condition); Biofilm: unique proteins in biofilm matrix of *H. somni*; OMV_Fe & OMV_EDDHA: proteins detected in OMVs under both iron-sufficient and deficient conditions. OMV_Fe & Biofilm: proteins detected in OMVs under iron-sufficient condition and in the biofilm matrix. OMV_EDDHA & Biofilm: proteins detected in OMVs under iron-deficient condition and in the biofilm matrix. Common: proteins detected under all three growth conditions tested in the study.

[b] The abundance shown here is the average of the relative quant of the designated protein measured in two replicates samples for one growth condition. We used the data for comparing the abundance of two different proteins under the same growth condition or the same protein between iron sufficient and deficient

conditions. Considering the concentration of biofilm proteins loaded for gel electrophoresis and proteomic analysis was not exactly as same as that of OMV proteins, we did not compare the abundance of the same protein between the biofilm group and the OMV groups.

[c] “No Quan Value” indicates that at least one data point of the abundance was missing for the corresponding protein. However, these proteins still had high FDR confidence and were considered present in the sample.

| **Supplemental Table S2. Sub-cellular localization of 92 common proteins in *H. somni* predicted through Psortb.** | | |
|---|---|---|
| **Protein ID** | **Localization** | **Score** |
| ACA31239.1 cysteine protease domain, YopT-type [*Histophilus somni* 2336] | Extracellular | 7.9 |
| ACA31627.1 Sel1 domain protein repeat-containing protein [*Histophilus somni* 2336] | Extracellular | 9.72 |
| DAA01283.1 TPA_exp: putative glycerophosphoryl diester phosphate | Extracellular | 8.99 |
| ACA31051.1 SmpA/OmlA domain protein [*Histophilus somni* 2336] | Outer Membrane | 9.93 |
| ACA31096.1 Peptidoglycan-associated lipoprotein [*Histophilus somni* 2336] | Outer Membrane | 9.93 |
| ACA31171.1 17 kDa surface antigen [*Histophilus somni* 2336] | Outer Membrane | 9.93 |
| ACA31193.1 porin Gram-negative type [*Histophilus somni* 2336] | Outer Membrane | 10 |
| ACA31204.1 outer membrane chaperone Skp (OmpH) [*Histophilus somni* 2336] | Outer Membrane | 9.93 |
| ACA31205.1 surface antigen (D15) [*Histophilus somni* 2336] | Outer Membrane | 10 |
| ACA31267.1 OmpA domain protein transmembrane region-containing protein [*Histophilus somni* 2336] | Outer Membrane | 10 |
| ACA31604.1 VacJ family lipoprotein [*Histophilus somni* 2336] | Outer Membrane | 9.93 |
| ACA31830.1 conserved hypothetical protein [*Histophilus somni* 2336] | Outer Membrane | 9.49 |
| ACA32113.1 Rare lipoprotein B [*Histophilus somni* 2336] | Outer Membrane | 9.92 |
| ACA32145.1 peptidase M23B [*Histophilus somni* 2336] | Outer Membrane | 8.86 |
| ACA32292.1 Tetratricopeptide TPR_2 repeat protein [*Histophilus somni* 2336] | Outer Membrane | 9.93 |
| ACA32396.1 Organic solvent tolerance protein [*Histophilus somni* 2336] | Outer Membrane | 10 |
| ACA32419.1 TonB-dependent lactoferrin and transferrin receptor [*Histophilus somni* 2336] | Outer Membrane | 10 |
| CBN71009.1 unnamed protein product [*Histophilus somni* 2336] | Outer Membrane | 10 |
| CBN71110.1 unnamed protein product [*Histophilus somni* 2336] | Outer Membrane | 10 |
| ACA30764.1 periplasmic binding protein/LacI transcriptional regulator [*Histophilus somni* 2336] | Periplasm | 9.76 |
| ACA30832.1 TRAP dicarboxylate transporter, DctP subunit [*Histophilus somni* 2336] | Periplasm | 9.76 |
| ACA30928.1 transport-associated [*Histophilus somni* 2336] | Periplasm | 9.44 |
| ACA30955.1 TRAP dicarboxylate transporter, DctP subunit [*Histophilus somni* 2336] | Periplasm | 9.76 |
| ACA30961.1 Monosaccharide-transporting ATPase [*Histophilus somni* 2336] | Periplasm | 9.76 |
| ACA30978.1 extracellular solute-binding protein family 3 [*Histophilus somni* 2336] | Periplasm | 10 |
| ACA30979.1 extracellular solute-binding protein family 1 [*Histophilus somni* 2336] | Periplasm | 9.76 |
| ACA31004.1 molybdopterin guanine dinucleotide-containing S/N-oxide reductase [*Histophilus somni* 2336] | Periplasm | 10 |
| ACA31013.1 outer membrane lipoprotein carrier protein LolA [*Histophilus somni* 2336] | Periplasm | 9.76 |
| ACA31015.1 protease Do [*Histophilus somni* 2336] | Periplasm | 9.76 |
| ACA31238.1 thiamine ABC transporter, periplasmic binding protein [*Histophilus somni* 2336] | Periplasm | 9.76 |
| ACA31288.1 extracellular solute-binding protein family 1 [*Histophilus somni* 2336] | Periplasm | 9.76 |
| ACA31337.1 extracellular solute-binding protein family 1 [*Histophilus somni* 2336] | Periplasm | 9.76 |
| ACA31508.1 multicopper oxidase type 3 [*Histophilus somni* 2336] | Periplasm | 10 |
| ACA31585.1 DSBA oxidoreductase [*Histophilus somni* 2336] | Periplasm | 9.76 |
| ACA31622.1 trimethylamine-N-oxide reductase TorA [*Histophilus somni* 2336] | Periplasm | 9.76 |
| ACA31787.1 periplasmic solute binding protein [*Histophilus somni* 2336] | Periplasm | 10 |

| | | |
|---|---|---|
| ACA31977.1 molybdenum ABC transporter, periplasmic molybdate-binding protein [*Histophilus somni* 2336] | Periplasm | 9.44 |
| ACA32073.1 Monosaccharide-transporting ATPase [*Histophilus somni* 2336] | Periplasm | 9.76 |
| ACA32358.1 extracellular solute-binding protein family 5 [*Histophilus somni* 2336] | Periplasm | 10 |
| ACA32460.1 extracellular solute-binding protein family 5 [*Histophilus somni* 2336] | Periplasm | 9.76 |
| ACA32483.1 extracellular solute-binding protein family 3 [*Histophilus somni* 2336] | Periplasm | 10 |
| ACA32487.1 periplasmic solute binding protein [*Histophilus somni* 2336] | Periplasm | 9.76 |
| ACA32496.1 periplasmic binding protein/LacI transcriptional regulator [*Histophilus somni* 2336] | Periplasm | 10 |
| ACA32601.1 Monosaccharide-transporting ATPase [*Histophilus somni* 2336] | Periplasm | 9.76 |
| ACA32669.1 extracellular solute-binding protein family 1 [*Histophilus somni* 2336] | Periplasm | 9.44 |
| sp\|B0UVZ0.1\|FTSP_HISS2 RecName: Full=Cell division protein FtsP; Flags: Precursor | Periplasm | 9.76 |
| sp\|B0UW71.1\|Y266_HISS2 RecName: Full=UPF0319 protein HSM_0266; Flags: Precursor | Periplasm | 9.84 |
| ACA30732.1 PpiC-type peptidyl-prolyl cis-trans isomerase [*Histophilus somni* 2336] | Cytoplasmic Membrane | 7.88 |
| ACA31314.1 carboxyl-terminal protease [*Histophilus somni* 2336] | Cytoplasmic Membrane | 9.99 |
| ACA32221.1 lipoprotein, YaeC family [*Histophilus somni* 2336] | Cytoplasmic Membrane | 9.97 |
| ACA30723.1 2-oxo-acid dehydrogenase E1 subunit, homodimeric type [*Histophilus somni* 2336] | Cytoplasm | 9.97 |
| ACA30724.1 pyruvate dehydrogenase complex dihydrolipoamide acetyltransferase [*Histophilus somni* 2336] | Cytoplasm | 9.97 |
| ACA30725.1 dihydrolipoamide dehydrogenase [*Histophilus somni* 2336] | Cytoplasm | 9.97 |
| ACA31418.1 ribosomal protein S16 [*Histophilus somni* 2336] | Cytoplasm | 9.26 |
| ACA31593.1 translation elongation factor G [*Histophilus somni* 2336] | Cytoplasm | 10 |
| ACA31758.1 ribosomal protein L14 [*Histophilus somni* 2336] | Cytoplasm | 9.97 |
| ACA31915.1 Beta-glucuronidase [*Histophilus somni* 2336] | Cytoplasm | 8.96 |
| ACA32007.1 ribosomal protein L1 [*Histophilus somni* 2336] | Cytoplasm | 9.26 |
| ACA32259.1 ribosomal protein S18 [*Histophilus somni* 2336] | Cytoplasm | 9.26 |
| ACA32447.1 ribosomal protein S15 [*Histophilus somni* 2336] | Cytoplasm | 9.26 |
| ACA32587.1 conserved hypothetical protein [*Histophilus somni* 2336] | Cytoplasm | 8.96 |
| sp\|B0USK6.1\|CH60_HISS2 RecName: Full=Chaperonin GroEL; AltName: Full=60 kDa chaperonin; AltName: Full=Chaperonin-60; Short=Cpn60 | Cytoplasm | 9.97 |
| sp\|B0USS4.1\|RS21_HISS2 RecName: Full=30S ribosomal protein S21 | Cytoplasm | 9.26 |
| sp\|B0UUZ6.1\|RL10_HISS2 RecName: Full=50S ribosomal protein L10 | Cytoplasm | 9.26 |
| sp\|B0UV21.1\|EFTU_HISS2 RecName: Full=Elongation factor Tu; Short=EF-Tu | Cytoplasm | 9.97 |
| sp\|B0UWR4.1\|DNAK_HISS2 RecName: Full=Chaperone protein DnaK; AltName: Full=HSP70; AltName: Full=Heat shock 70 kDa protein; AltName: Full=Heat shock protein 70 | Cytoplasm | 9.97 |
| sp\|B0UX31.1\|RS5_HISS2 RecName: Full=30S ribosomal protein S5 | Cytoplasm | 9.26 |
| sp\|B0UX40.1\|RL17_HISS2 RecName: Full=50S ribosomal protein L17 | Cytoplasm | 9.26 |
| ACA30959.1 ABC-type sugar-binding periplasmic protein [*Histophilus somni* 2336] | Unknown | 2.5 |
| ACA31071.1 conserved hypothetical protein [*Histophilus somni* 2336] | Unknown | 2.5 |
| ACA31348.1 toluene tolerance family protein [*Histophilus somni* 2336] | Unknown | 2.5 |
| ACA31465.1 conserved hypothetical protein [*Histophilus somni* 2336] | Unknown | 2.5 |

| | | |
|---|---|---|
| ACA31502.1 protein of unknown function DUF411 [*Histophilus somni* 2336] | Unknown | 2.5 |
| ACA31613.1 protein of unknown function DUF302 [*Histophilus somni* 2336] | Unknown | 2.5 |
| ACA31633.1 NADH:ubiquinone oxidoreductase, subunit C [*Histophilus somni* 2336] | Unknown | 2.5 |
| ACA31714.1 sugar ABC transporter, sugar-binding protein [*Histophilus somni* 2336] | Unknown | 2.5 |
| ACA31740.1 ABC transporter, solute-binding, sugar transport [*Histophilus somni* 2336] | Unknown | 2.5 |
| ACA31804.1 conserved hypothetical protein [*Histophilus somni* 2336] | Unknown | 2 |
| ACA31914.1 conserved hypothetical protein [*Histophilus somni* 2336] | Unknown | 2.5 |
| ACA31970.1 conserved hypothetical protein [*Histophilus somni* 2336] | Unknown | 2 |
| ACA31971.1 conserved hypothetical protein [*Histophilus somni* 2336] | Unknown | 2 |
| ACA31973.1 conserved hypothetical protein [*Histophilus somni* 2336] | Unknown | 2.5 |
| ACA31980.1 conserved hypothetical protein [*Histophilus somni* 2336] | Unknown | 2.5 |
| ACA32200.1 conserved hypothetical protein [*Histophilus somni* 2336] | Unknown | 2.5 |
| ACA32521.1 hypothetical protein HSM_0848 [*Histophilus somni* 2336] | Unknown | 2.5 |
| ACA32634.1 SmpA/OmlA domain protein [*Histophilus somni* 2336] | Unknown | 2 |
| ACA32668.1 extracellular solute-binding protein family 1 [*Histophilus somni* 2336] | Unknown | 2.5 |
| sp\|B0UTT2.1\|LPOA_HISS2 RecName: Full=Penicillin-binding protein activator LpoA; Short=PBP activator LpoA; Flags: Precursor | Unknown | 2 |
| ACA31503.1 multicopper oxidase type 3 [*Histophilus somni* 2336] | Unknown* | 5.41 |
| ACA32109.1 PpiC-type peptidyl-prolyl cis-trans isomerase [*Histophilus somni* 2336] | Unknown* | 5.81 |
| ACA32359.1 extracellular solute-binding protein family 5 [*Histophilus somni* 2336] | Unknown* | 5.81 |
| CAY37763.1 unnamed protein product [*Histophilus somni* 2336] | Unknown* | 4.99 |

**Supplemental Table S3: Unique and common proteins categorized by their KEGG pathways.**

| **Group [a]** | **Protein ID** | **Gene locus tag** | **KEGG category [b]** | **KEGG pathway [c]** |
|---|---|---|---|---|
| OMV_Fe | ACA31054.1 | HSM_0132 | | |
| OMV_Fe | B0UVM9.1 | HSM_0168 | G | Ribosome |
| OMV_Fe | ACA32015.1 | HSM_0377 | | |
| OMV_Fe | ACA32034.1 | HSM_0394 | | |
| OMV_Fe | ACA32047.1 | HSM_0405 | | |
| OMV_Fe | ACA32075.1 | HSM_0430 | E | ABC transporters |
| OMV_Fe | ACA30734.1 | HSM_1022 | | |
| OMV_Fe | ACA31654.1 | HSM_1864 | | |
| OMV_Fe | ACA31752.1 | HSM_1958 | G | Ribosome |
| OMV_Fe | B0UX32.1 | HSM_1971 | G | Ribosome |
| OMV_EDDHA | ACA32586.1 | HSM_0906 | | |
| OMV_EDDHA | ACA32592.1 | HSM_0911 | | |
| OMV_EDDHA | ACA32614.1 | HSM_0931 | | |
| OMV_EDDHA | ACA32615.1 | HSM_0932 | | |
| OMV_EDDHA | ACA30786.1 | HSM_1071 | H, M | Metabolic pathways; beta-Lactam resistance; Peptidoglycan biosynthesis |
| OMV_EDDHA | ACA31186.1 | HSM_1440 | | |
| OMV_EDDHA | ACA31510.1 | HSM_1732 | | |
| Biofilm | ACA31798.1 | HSM_0002 | G | DNA replication; Mismatch repair; Homologous recombination |
| Biofilm | B0UUW5.1 | HSM_0007 | M | Pyrimidine metabolism; Metabolic pathways |
| Biofilm | ACA31031.1 | HSM_0013 | M | Valine, leucine and isoleucine biosynthesis; Butanoate metabolism; C5-Branched dibasic acid metabolism; Pantothenate and CoA biosynthesis; Metabolic pathways; Biosynthesis of secondary metabolites; 2-Oxocarboxylic acid metabolism; Biosynthesis of amino acids |
| Biofilm | ACA31140.1 | HSM_0014 | M | Valine, leucine and isoleucine biosynthesis; Butanoate metabolism; C5-Branched dibasic acid metabolism; Pantothenate and CoA biosynthesis; Metabolic pathways; Biosynthesis of secondary metabolites; 2-Oxocarboxylic acid metabolism; Biosynthesis of amino acids |
| Biofilm | ACA31843.1 | HSM_0022 | C; E; G | Quorum sensing; Protein export; Bacterial secretion system |
| Biofilm | ACA31932.1 | HSM_0030 | G | Ribosome |
| Biofilm | CAV20865.1 | HSM_0032 | G | Fatty acid biosynthesis; Metabolic pathways; Fatty acid metabolism |
| Biofilm | ACA31963.1 | HSM_0033 | M | Fatty acid biosynthesis; Metabolic pathways; Biosynthesis of secondary metabolites; Fatty acid metabolism |
| Biofilm | CBG07608.1 | HSM_0034 | | |
| Biofilm | B0UUZ3.1 | HSM_0035 | M | Metabolic pathways; Biosynthesis of secondary metabolites |
| Biofilm | ACA31996.1 | HSM_0036 | G | Ribosome |
| Biofilm | ACA32029.1 | HSM_0039 | G | Ribosome |
| Biofilm | ACA32041.1 | HSM_0040 | G | RNA degradation |
| Biofilm | ACA32063.1 | HSM_0042 | M | Pentose phosphate pathway; Pentose and glucuronate interconversions; Metabolic pathways; Biosynthesis of secondary metabolites; Microbial |

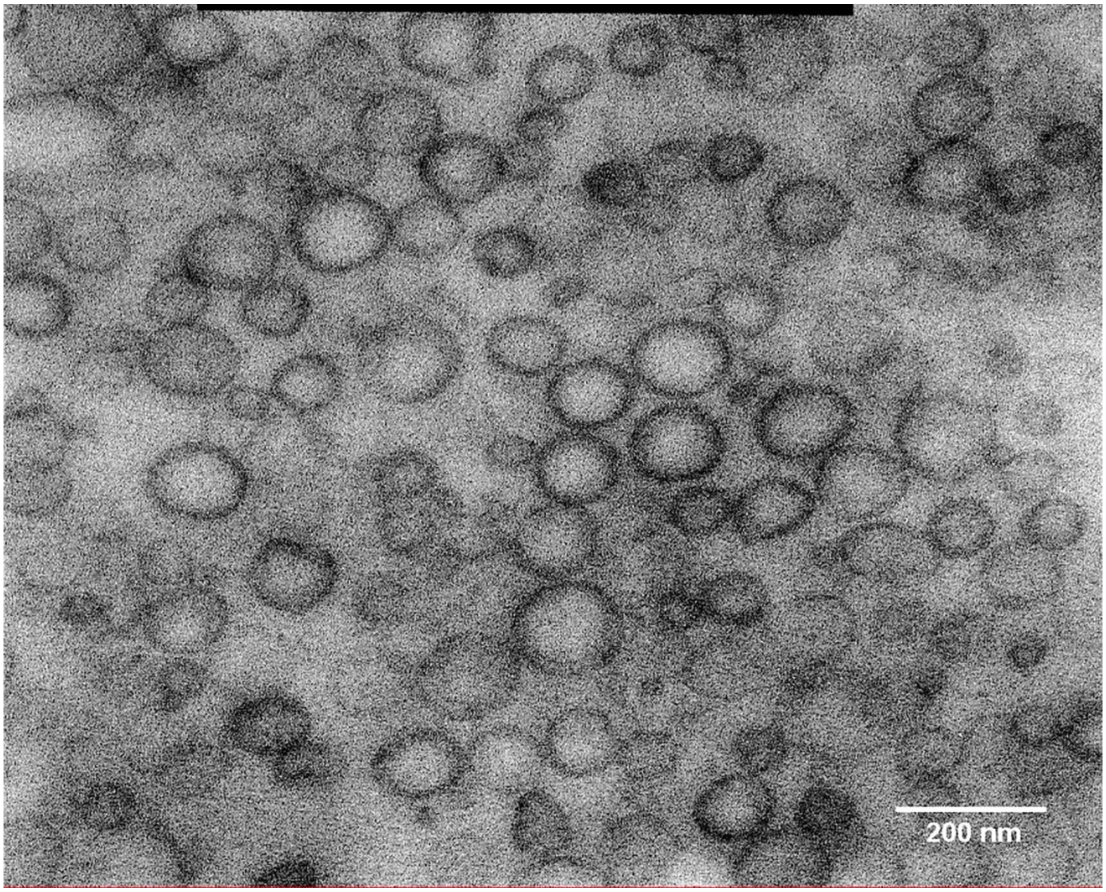


**Supplemental Fig. S1. Outer membrane vesicles (OMV) of *H. somni*.** OMV from cell-free culture supernatant were pelleted by centrifugation, resuspended in 0.3 ml of 2.5% glutaraldehyde, and processed for TEM at the Cold Spring Harbor Shared Microscopy Resource. Magnification 50,000 x.

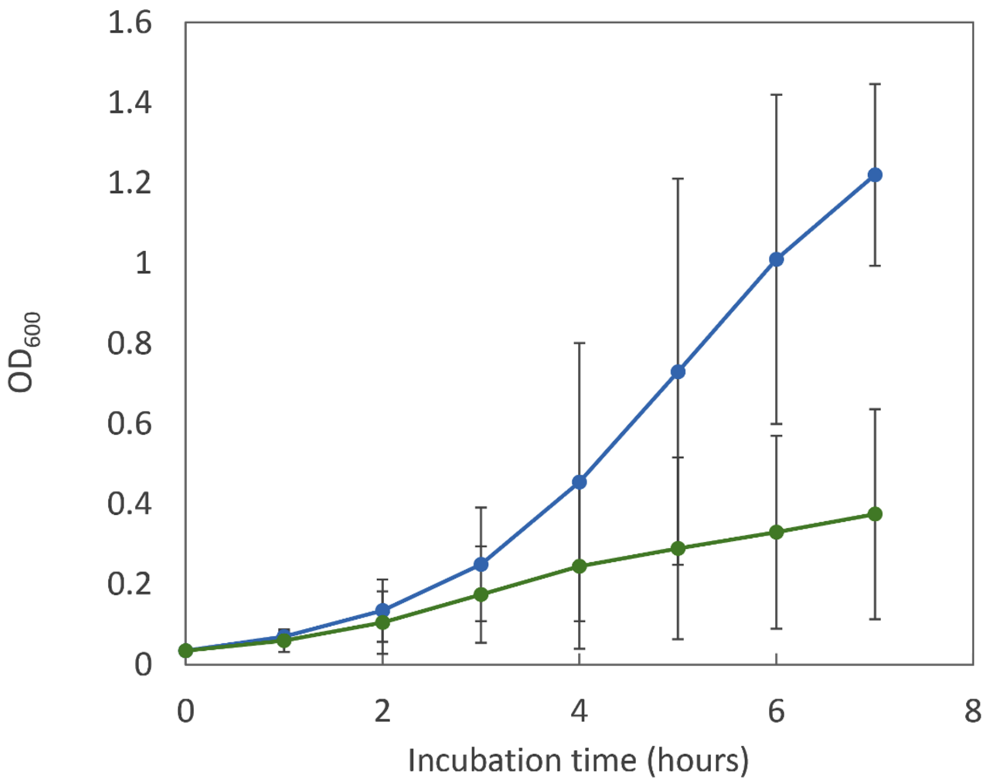


**Supplemental Fig. S2. Growth curve of *H. somni* in BYTT with or without EDDHA.** *H. somni* from a fresh overnight plate of growth was resuspended in PBS to an $OD_{600}$ of 0.8 (about $10^9$ CFU/ml). Bacteria were diluted 1 to 20 with 8 ml of BHI broth supplemented with 0.5% yeast extract, 0.1% Trizma base, and 0.01% TMP (BYTT), resulting in an $OD_{600}$ of 0.02-0.04. The cultures were transferred to 2 tubes containing 4 ml of BYTT each. One of the paired tubes was supplemented with a 1:50 dilution of filter-sterilized 10 mM EDDHA (——), and the other with ferric nitrate (——). Bacteria were incubated at 37°C with shaking at 200 rpm. The $OD_{600}$ of the cultures was measured hourly.
